# Towards a cleaner CHO chassis: systematic knockout of host cell proteins for efficient biopharmaceutical manufacturing

**DOI:** 10.3389/fbioe.2026.1750646

**Published:** 2026-01-30

**Authors:** Jannis Peter Marzluf, Jennifer Klein, Daniela Kirchmeier, Alexander Stärk, Daniel Ziegner, Markus Gehrung, Franziska Rauh, Merle Rattay, Christoph Zehe, Ann-Cathrin Leroux

**Affiliations:** 1 Corporate Research, Sartorius Stedim Cellca GmbH, Ulm, Germany; 2 Department of Gene Therapy, University of Ulm, Ulm, Germany

**Keywords:** biopharmaceuticals, bioprocessing, cell line development (CLD), Chinese hamster ovary cells (CHO), host cell protein (HCP), multiplex gene editing

## Abstract

Host cell proteins (HCPs) are major process‐related impurities in biopharmaceutical manufacturing, imposing metabolic burden on production hosts and complicating downstream purification, thereby impacting cost, product stability, and patient safety. Here, we engineered Chinese hamster ovary (CHO) cells toward a streamlined production chassis through multiplex CRISPR‐mediated knockout of abundant and difficult‐to‐remove HCPs. Proteomic profiling across multiple clones, products, and downstream purification steps identified a core set of highly abundant proteins that contributed disproportionately to total released HCP mass and showed limited removal by standard purification processes. Screening of 62 candidate genes using pooled and single‐cell knockout approaches identified targets compatible with cell growth and productivity, yielding 36 validated candidates suitable for host‐cell streamlining. Multiplex editing of selected targets achieved approximately 57–85% reduction in released HCP levels, depending on configuration, without compromising growth, viability, or productivity in fed‐batch cultures. The resulting cell lines maintained bioprocess performance while substantially lowering impurity load. Together, this work demonstrates a scalable, proteomics‐guided strategy for rational host‐cell engineering that enables upstream reduction of persistent impurities and supports cleaner, safer, and more cost‐efficient biopharmaceutical manufacturing.

## Introduction

Chinese hamster ovary (CHO) cells remain the workhorse for therapeutic protein manufacturing. The vast majority of approved biologics are produced in this host system due to the adaptability to large-scale culture, capacity for high volumetric productivity, and ability to generate human-like post-translational modifications within a regulatory framework built on decades of use (Tihanyi and Nyitray, 2020; Kim et al., 2012; Walsh and Walsh, 2022; Majumdar et al., 2024). Despite continuous improvements in culture media and process design, there is persistent demand for host cell engineering strategies that further increase productivity, stabilize expression, and introduce beneficial process or product traits (Ha et al., 2020; Karottki et al., 2021; Amiri et al., 2023; Kol et al., 2020; Weiß et al., 2025; Lin et al., 2021; Kim et al., 2024; Xiong et al., 2021; Marzluf et al., 2025a).

The advent of CRISPR/Cas9-based editing, together with systems-level analyses from transcriptomics, metabolomics, and proteomics, has provided new opportunities to rationally redesign CHO cells (Amiri et al., 2023). Targeted genetic modifications have been shown to delay apoptosis (Ha et al., 2020; Grav et al., 2015; Tang et al., 2022), improve product stability (Weiß et al., 2025; Chiu et al., 2017; Laux et al., 2018), and eliminate antibody fucosylation (Yamane‐Ohnuki et al., 2004), thereby improving antibody dependent cytotoxicity (Peipp et al., 2008). Additional studies have demonstrated that genome engineering can elevate secretion capacity (Hansen et al., 2017; Lu et al., 2018) and lower host cell protein (HCP) levels (Kol et al., 2020; Fukuda et al., 2019). Hybrid CRISPR/RMCE strategies have further enabled targeted, modular redesign of CHO cells for improved productivity and phenotype control (Adibzadeh et al., 2024).

HCPs represent a persistent and costly challenge in biomanufacturing (Li, 2017; Ho et al., 2014). Even trace levels of co-purified HCPs can compromise product quality (Chiu et al., 2017; Laux et al., 2018) or patient safety (Fischer et al., 2017; Wang et al., 2009), prompting stringent purification requirements that increase process complexity and cost (Kol et al., 2020; Jones et al., 2021). Lipoprotein lipase (LPL) is one well characterized example: it is difficult to remove during purification and catalyzes polysorbate degradation, thereby reducing shelf life of antibody formulations (Chiu et al., 2017). Further recent work on polysorbate degrading enzymes also demonstrated that multiplex knockout of lipase and hydrolase genes can substantially mitigate polysorbate degradation, providing a genetic upstream solution to this long-standing biologics storage challenge (Weiß et al., 2025).

Multi-step downstream processing accounts for a majority of total antibody mAb manufacturing costs (Guiochon and Beaver, 2011), with typical IgG-Formats going through Protein A capture followed by virus inactivation and polishing steps such as cation-exchange and anion-exchange chromatography (Shukla and Thömmes, 2010). While these steps remove the bulk of contaminants, specific HCPs can escape all steps and persist in final drug substance, where they may retain enzymatic activity, impact product stability and patient safety (Fischer et al., 2017; Goey et al., 2018). Monitoring of total HCP levels in industry typically relies on enzyme-linked immunosorbent assays (ELISA), which provide aggregate concentration data but no information on the identity or properties of individual proteins (Zhu-Shimoni et al., 2014). In contrast, proteomic approaches such as LC–MS/MS enable direct characterization of specific HCPs, revealing those that are difficult to clear or detrimental to product quality (Kol et al., 2020; Valente et al., 2018; Kumar et al., 2015).

Genetic removal of abundant or difficult-to-remove HCPs represents a powerful upstream solution as demonstrated by recent studies. Kol et al. (2020) systematically applied multiplex CRISPR editing to eliminate up to 14 abundant or difficult-to-remove HCPs, achieving 40%–70% reductions in total HCP levels. While the highest-order knockout clones (11× and 14×) showed reduced productivity compared with their wild-type counterparts, stable pools and selected single-cell clones demonstrated maintained titers, indicating that productivity impacts depend strongly on the specific KO configuration and clonal background. Building on this work, we systematically identified HCP genes that are both abundant, energetically costly to produce, as well as those persisting through downstream purification. Using mass-spectrometry–based profiling, we prioritized candidate HCPs and evaluated knockout feasibility in both single-cell clones and our previous established high-throughput pooled KO screening platform (Marzluf et al., 2025b) under standard fed-batch conditions in an industrially established CHO production platform. With validated targets, we applied sequential multiplex gene editing to generate CHO cell lines carrying up to eleven simultaneous HCP knockouts, enabling evaluation of stepwise HCP removal on growth, viability, productivity, and HCP levels. These engineered host cells showed cumulative HCP reductions ranging from ∼15% to ∼85% with no negative impact on bioprocess performance.

## Materials and methods

### Cell lines and cultivation conditions

CHO DG44 cells (female) were obtained from Lawrence Chasin (Columbia University) and adapted in-house for serum-free suspension growth in chemically defined media. Cells were cultured in serum- and antibiotics-free CD DG44 medium (Gibco™) supplemented with 6 mM L-glutamine (Sartorius) and 0.1% Pluronic F-68 (Gibco™). The host cell was cultivated in 50 mL in 250 mL shake-flasks (Corning, NY, United States) at 36.8 °C, 7.5% CO_2_, and 80% humidity and passaged every 2 days to an inoculation density of 2 × 10^5^ cells/mL. This host cell served as the starting point for all experiments. Following transfection, cells were cultivated sequentially in 24-well plates (1 mL; Nunc), 6-well plates (4 mL; Nunc), and 125 mL shake-flasks (25 mL; Corning) for cell banking and experimentation. Cell density and viability were monitored using CASY® Cell Counter and Analyzer (OMNI Life Science). Cells were routinely tested and confirmed negative for *mycoplasma* contamination.

### Downstream processing (DSP) conditions

Harvest supernatants were clarified and purified through a standard platform sequence consisting of Protein A affinity capture, low-pH virus inactivation, cation-exchange (CEX), and anion-exchange (AEX). Purification and sample collection after each step were performed externally (Sartorius) under platform SOPs, key set-points (resins, load densities, residence times, buffers, pH and conductivity) were fixed across clones for comparability but are not disclosed here. Aliquots from ProtA, VI, CEX, and AEX were submitted to Alphalyse A/S for SWATH LC–MS analysis and HCP identification/quantification.

### Identification of HCPs by mass spectrometry

Fed-batch supernatants were harvested from multiple CHO production clones expressing IgG1 molecules under standard and varied process conditions. Samples were centrifuged to remove cell debris and clarified supernatants were analyzed by peptide mapping LC–MS/MS (Alphalyse). Proteins were identified against a manually curated reference proteome of *Cricetulus griseus* from the UniProt database (Consortium, 2018), and annotations for secretion signals and subcellular localization were retrieved from UniProt and confirmed with PrediSi (Hiller et al., 2004). Temporal profiling was conducted by collecting supernatant samples up to 5 days prior to harvest. Additional samples taken after individual downstream processing (DSP) steps, Protein A affinity capture, low pH virus inactivation, cation exchange (CEX), and anion exchange (AEX) chromatography, were subjected to the same LC–MS/MS analysis to assess HCP persistence through purification. Under the applied LC–MS/MS workflow, HCP detection was qualitative to semi-quantitative, with a practical detection limit in the low ng/mL range for secreted proteins, depending on peptide ionization efficiency and sample complexity. “Core” HCPs, defined as proteins consistently detected across clones and purification steps, were determined by Venn analysis (Draw Venn Diagram). Translation and secretion energy requirements were estimated following published approaches (Kol et al., 2020). The energetic investment per secreted protein was calculated as:

ATP_cost = TPM × [ 4 L (translation) + 22 (signal peptide degradation) + (L/40 + 2) (ER translocation)], where L is the amino-acid length of the protein. This formulation estimates the relative energetic burden associated with protein synthesis and secretion under steady-state conditions, assuming proportionality between transcript abundance and protein production. It does not account for intracellular protein turnover, degradation rates, or differences in secretion efficiency between proteins. Transcript-level abundances (TPM) were obtained from RNA-seq of production cultures and used alongside MS-derived extracellular abundance data to contextualize relative energetic investment rather than absolute cellular ATP consumption.

### Synthetic sgRNA design and CRISPR/Cas9 RNP transfection

Gene targets for this study are shown in Supplementary Table S1. Guide RNA sequences were designed using Geneious Prime software, version 11.0.11 (Biomatters) or the CRISPR Guide RNA Design software (https://www.benchling.com) targeting an early exon present in all transcript variants to induce early frameshift mutations, rendering the translated protein unfunctional. gRNAs were selected based on scoring on-target efficiency (Doench et al., 2016) and off-target specificity (Hsu et al., 2013). It was ensured that possible off-targets reside in non-protein-coding sequences of the host genome. Off-target scoring was done against the fully sequenced and annotated host cell genome. Three sgRNAs were screened for each target gene to use the highest efficiency sgRNA in downstream experimentation. sgRNAs were ordered as TrueGuide™ Synthetic gRNAs at ThermoFisher Scientific (Thermo Fisher Scientific) and transfected with TrueCut™ Cas9 Protein v2 (Thermo Fisher Scientific) in 1:1 ratio as 7.5 pmol pre-assembled RNP complexes (Amiri et al., 2025) into 2E5 cells using a NEON™ Transfection System and NEON™ Transfection System 10 µL Kit (Thermo Fisher Scientific). Transfection parameters were set to 1700 V, 20 ms pulse width and 1 pulse. The RNP transfected host cells were subjected to genotyping by genomic DNA extraction, PCR and Sanger-sequencing analysis followed by ICE-analysis 48 h after transfection as described below.

### Genotyping and KO verification

Genomic DNA was extracted from transfected pools or single cell clones using the QuickExtract™ DNA Extraction Solution (Lucigen), followed by PCR to amplify the genomic regions flanking the sgRNA cut site aiming for 200–300 bp up- and downstream. Amplicons were sent for PCR purification and Sanger sequencing at Microsynth Seqlab GmbH (Göttingen). The Sanger sequencing traces for each test sample (edited) and its corresponding control sample (unedited) were uploaded to the Inference of CRISPR Edits (ICE) software tool, version 3.0 (ice.synthego.com) (Synthego) and analyzed according to the developer’s instructions. ICE analysis provides the InDel percentage and the KO score. The InDel percentage reflects the editing efficiency of the edited trace compared to the control trace, irrespective of whether the InDel results in a frameshift. The KO score indicates the KO efficiency, representing the proportion of cells with either a frameshift InDel or a fragment deletion, which is likely to result in a functional knockout. The gRNA with the highest KO score for a given gene target was selected for high editing efficiency and a favorable InDel profile, characterized by predominantly out-of-frame InDels and a high KO score, with a preference for sgRNAs that produce a limited variety of InDels, ideally a single dominant type.

### Generation of KO clones and high KO proportion pools

To generate KO populations of the CHO DG44 host cell for all target genes, transfections were performed using the previously selected best sgRNA out of the three screened sgRNAs. These single RNP transfected pools were subsequently used for single cell cloning to derive KO clones for further experimentation. Single cell clones were generated using high-throughput nanowell-based image-verified cloning with the CellCelector (Sartorius). Each well of a 24-nanowell plate was filled with proprietary 0.5 mL cloning medium. The plate was centrifuged (800 × g, 5 min) to fully settle the media into the bottom grid of the nanowells ensuring full liquid contact. A total of 2000 cells were seeded into these wells by diluting the target cells to 4000 cells/mL in cloning medium and seeding 0.5 mL of this dilution into one nanowell. Ahead of seeding, the sample was passed through a cell strainer (Corning). Each of the 24-wells contains approximately 4400 nanowells, resulting in <0.5 cells being seeded per well. At the end of seeding the plate was again centrifuged (300 × g, 3 min) to settle all cells. Cells are scanned using the CellCelector (Sartorius) on the day of seeding to ensure monoclonality and then again after 4 days to assess growth and pick outgrown clones (15–20 cells) into 384 well-plate containing standard cultivation media. Monoclonality was image-verified at seeding (day 0). Clones were accepted if a single starting cell and continuous growth were documented. Confluency of clones was measured using the CellMetric (Solentim) intermittently from days seven to fourteen. Once most of the clones reached 70%–100% confluency, all clones were transferred to 96-well plate and passaged every 3–4 days while genotyping took place. Full KO-clones were expanded to 12-well plates in 1.5 mL standard cultivation media before expansion to 6-well and shake flask under standard conditions.

For the generation of KO pools with high KO scores the same RNP transfection was performed three sequential times 48 h apart with the same cells. These pools were then expanded for further experimentation. Pools required a pre-inoculation KO-score ≥66% (ICE). KO-scores were measured at sgRNA validation, post-sequential transfections, at fed-batch day 0, and day 7. Targets with absolute KO-score drift >10% between validation and inoculation were excluded from phenotypic analysis.

For multiplex KO generation, preassembled Cas9–sgRNA ribonucleoprotein (RNP) complexes for multiple targets were combined into a single transfection mix and introduced simultaneously into CHO DG44 cells using the Neon system. Each sgRNA was complexed with Cas9 at a 1:1 M ratio, and the total RNP amount was either evenly distributed among targets or scaled proportionally depending on the number of guides, as optimized in preliminary multiplexing experiments. Serial transfections were performed three times at 48-h intervals to increase overall editing efficiency.

### Fed-batch characterization of KO pools and clones

Stable producer pools were generated by transfecting each KO pool or clone with an IgG1 expression plasmid, followed by antibiotic selection and adaptation in 4Cell® SmartCHO medium (Sartorius) until stable growth and viability were achieved. Five clonal and seven pooled screening batches were executed. Each batch contained an unedited WT production pool processed in parallel and used for within-batch normalization. Transfected cells were adapted in 4Cell® SmartCHO Stock and Adaptation medium (Sartorius) until growth and viability were restored. The resulting producer pools were then used to inoculate fed-batch cultures at 3 × 10^5 viable cells/mL in 25 mL 4Cell® SmartCHO PM medium (Sartorius) supplemented with 6 mM glutamine, cultivated in 125 mL shake flasks (Corning). Glucose and lactate concentrations in culture supernatants were measured using a Biosen C-Line analyzer (EKF Diagnostics), employing an enzymatic amperometric chip-sensor method. The analyzer was calibrated daily using certified standards, and quality control samples were run in parallel to verify precision. Cultures were fed with 4Cell® SmartCHO FMA (Sartorius) and 4Cell® SmartCHO FMB (Sartorius). The glucose concentration was adjusted to 5 g/L in addition to feeding. After 14 days or once viability reached below 70% the culture was harvested to collect supernatant. Antibody titer was quantified using the Octet® HTX system (Sartorius). The antibody concentration of retention samples was calculated using a standard curve of the purified antibody.

### Quantification of HCP by Octet ELISA

Total HCP was measured using the Anti-CHO HCP Detection Kit (Sartorius, Cat. No. 18–5158) on the Octet HTX platform (Sartorius), following the manufacturer’s protocol. A 7-point standard curve (3.13–200 ng/mL) was included on each plate, and samples were diluted 1:7,500 in two steps to fall within the linear range (*R* ^2^ > 0.99). Standards and samples were run in technical duplicates, and data were analyzed using Octet® Analysis Studio v12.2.2.26 (Sartorius). HCP concentrations were converted to parts per million (ppm) relative to product as: ppm HCP = (HCP concentration ÷ product concentration) × 1,000,000 using Octet Protein A titer measurements from parallel samples.

### Targeted LC–MS/MS for KO verification

For KO verification, harvest supernatants from selected KO clones and a WT control were analyzed by Alphalyse A/S using a standardized LC–MS/MS workflow. Retention samples were clarified (6.600 × g, 23 °C, 5 min), supernatants aliquoted (1 mL), stored at −20 °C, and shipped on dry ice. Protein concentrations were determined prior to shipment using anti-CHO HCP ELISA (Octet HTX) (Sartorius) and Protein A biosensors (Octet HTX)(Sartorius) to ensure the required ∼1 mg total protein per sample. Sample preparation followed Alphalyse’s automated protocol: (i) spike-in of seven standard proteins at known concentrations, (ii) protein precipitation to remove DNA and interfering substances, (iii) re-solubilization and tryptic digestion (16 h, 37 °C, enzyme:substrate ratio 1:50). Peptides were analyzed on a high-resolution LC–MS/MS platform using a combination of data-dependent acquisition (IDA/DDA, three runs) to build spectral ion libraries, and data-independent SWATH acquisition (two runs) for reproducible quantification. Raw data were searched against a CHO + IgG + contaminants UniProt database (Consortium, 2018) with a peptide- and protein-level false discovery rate (FDR) of 1%. Identification required at least one unique proteotypic peptide per protein. Presence/absence and relative abundance of the seven KO targets (BGN, FN1, LPL, NID1, PCOLCE, PXDN, THBS1) were assessed by monitoring target-specific proteotypic peptides. In cases of borderline detection, targeted confirmation by PRM/SRM was applied. Results were reported as absolute HCP concentrations (ng/mL), relative abundances, and in ppm relative to IgG concentration.

### Statistical analysis

Data are shown as mean ± standard deviation (SD). Statistical testing was performed in GraphPad Prism (Dotmatics, v10.6.1). Ordinary one-way or two-way ANOVA was used for comparisons involving more than two groups, and two-tailed unpaired t-tests for two-group comparisons. Significance was accepted at *P* < 0.05. The number of biological and technical replicates is specified in the figure legends.

Each knockout screening run included an unedited wild-type (WT) production pool, which served as an internal control. To correct for batch effects, all process metrics were normalized to the run-specific WT mean, yielding fold-change values used in hit calling and ranking analyses. Performance cutoffs were defined at approximately ±2× the WT standard deviation observed across replicates in each run (exact values in Supplementary Figure S7A). Knockouts falling outside the lower bound were classified as negative for that metric, those within the range were considered WT-like, and those above the upper bound were flagged as potentially beneficial. KO pools were excluded from analysis if KO scores declined by more than 10% absolute between validation and inoculation, or if the KO efficiency was <66%. Cultures that failed for technical reasons were omitted. Correlations among process metrics were computed on raw, non-normalized values pooled across runs, and reported as Pearson *r*. Heatmaps and pairwise scatterplots were generated in Python (Python Software Foundation, 2024) (v3.13.2) using packages pandas (McKinney, 2010) (v2.2.2), numpy (Harris et al., 2020) (v1.26.2) and matplotlib (Hunter, 2007) (v3.9.2) with pool and clone experiments distinguished by color. UpSet analyses were performed in R (R Core Team, 2024) (v4.4.2) ComplexUpset (Krassowski, 2023) (v1.3.3), where each gene was scored according to whether it passed or failed performance cutoffs across the four key metrics (IVCC, process time, final titer, mean Qp). Genes with unstable KO scores were excluded from this intersection analysis. Bar plots, rank plots, and compact composites were produced in Python (Python Software Foundation, 2024) (v3.13.2), while UpSet plots were generated in R (R Core Team, 2024) (v4.4.2).

## Results

### Concentrated burden: few HCPs are highly abundant and difficult to remove

To rationally select HCPs for KO, we characterized the extracellular proteome of a broad range of production clones, expressed products and process conditions. The objective was to identify common proteins that are regularly observed in culture supernatants and impose a substantial metabolic burden on the cell. Highly abundant HCPs were identified by analyzing 27 supernatant samples from fed-batch cultivations in Ambr250®-fed-batch cultures encompassing five clones (C1–C5) producing two different IgG1 recombinant proteins (A and B), under both standard and varied process conditions (Table 1). The larger number of samples for product B reflects its use as a process-variation reference clone, whereas product A was represented by fewer clones under matched harvest conditions to support cross-product comparisons rather than statistical equivalence. Additional samples were collected up to 5 days before harvest to capture temporal dynamics in HCP release by secretion and lysis. Following centrifugation, supernatants were subjected to LC–MS/MS peptide mapping analysis (Alphalyse, Denmark). In total, 1,254 unique HCP species were detected across all samples, products and conditions. Approximately ∼60% of species were consistently observed throughout the production phase in individual clones, indicating that most secreted proteins appear well before a decline in cell viability before notable cell lysis has occurred (Supplementary Figure S1A). Clones originating from the same transfection pool displayed ∼71% overlap in detected HCP species (Supplementary Figure S1B), indicating that clonal derivation contributes to extracellular proteome composition. Similarly, clones producing the same recombinant product shared 64% of their detected HCP species, whereas cross-product comparisons showed ∼67% overlap (Supplementary Figure S1C), highlighting an influence of the clone and expressed product on the released HCP profile. Process conditions also had an impact on HCP profiles: A single clone cultivated under eight different bioprocess settings retained just ∼53% of its HCP species across all conditions (Supplementary Figure S1D). Overall, the number of identified HCPs per sample ranged from ∼500 up to ∼800 species (Supplementary Figure S1E).

**TABLE 1:** Sample Overview for Peptide Mapping LC-MS/MS. All samples were taken at the harvest day, additional samples were taken 5, 4, 3, 2 or 1 day before harvest and are denoted as H-n. C1-3 originate from the same cell pool. C5 in the standard process was performed as biological replicate.

Endpoint harvest samples					Additional samples at				
Clone	Product	Process	Harvest day	Viability at harvest [%]	H-5	H-4	H-3	H-2	H-1
C1	A	Standard	12	60	​	​	​	​	​
C2			12	60	​	x	x	x	x
C3			14	60	​	​	​	​	​
C4			12	80	x	x	​	x	x
C5	B	Standard	12	84	​	x	x	x	x
			12	84	​	​	​	​	​
			12	81	​	​	​	​	​
		Process 1	12	84	​	​	​	​	​
		Process 2	12	74	​	​	​	​	​
		Process 3	12	84	​	​	​	​	​
		Process 4	12	85	​	​	​	​	​
		Process 5	12	85	​	​	​	​	​
		Process 6	12	85	​	​	​	​	​
		Process 7	12	80	​	​	​	​	​
		Process 8	12	74	​	​	​	​	​

**FIGURE 1:**
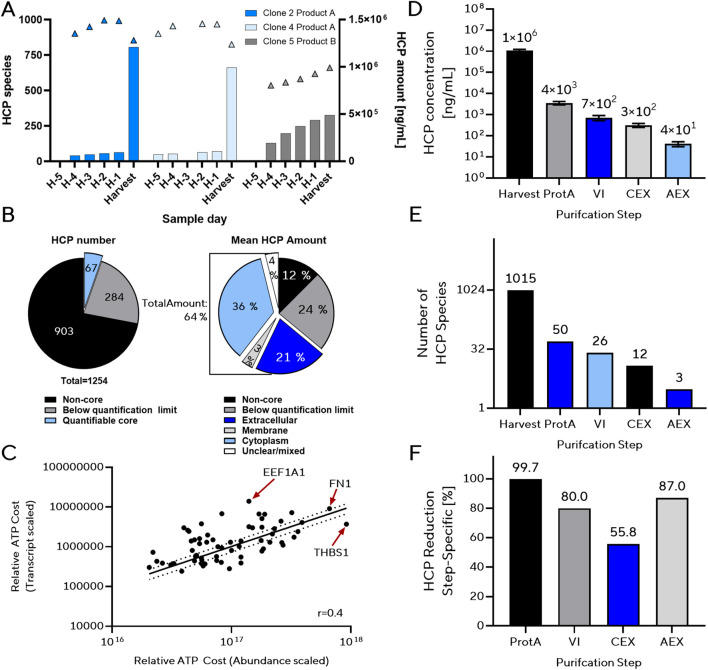
Identification of abundant and difficult-to-remove host cell proteins (HCPs) in the CHO production platform. **(A)** Temporal HCP profiles for representative clones producing two different IgG1 products under standard fed-batch conditions. Triangles (left axis) indicate the number of detected HCP species, and Bars (right axis) indicate total HCP concentrations at each pre-harvest sampling day (H–n) and at harvest. Clone 5 H-5 sample has failed and therefore is not applicable. **(B)** Composition of the CHO extracellular proteome. *Left*: proportion of total HCP species assigned to the core set (detected across all clones, products, and process conditions), including those consistently quantifiable (quantifiable core, n = 67). *Right*: distribution of mean HCP mass across subcellular localizations within the quantifiable core. **(C)** Correlation between transcript-based and extracellular abundance–based energetic cost estimates for quantifiable core HCPs. Relative ATP cost per protein species was calculated from amino acid length, secretion costs, and transcript abundance (TPM) or extracellular amount (MS-based). Pearson correlation r = 0.40, *R* ^2^ = 0.16, p < 0.001. Key abundant, costly proteins are labeled. **(D)** Absolute HCP concentrations at each downstream processing (DSP) stage: Protein A affinity chromatography (ProtA), virus inactivation (VI), cation exchange chromatography (CEX), and anion exchange chromatography (AEX). **(E)** Reduction in the number of distinct HCP species across DSP steps. **(F)** Step-specific HCP clearance efficiencies in total amount, expressed as percentage reduction relative to the preceding step.

Analysis of pre-harvest samples showed a trend in which the number of detected HCP species generally increased toward harvest (Figure 1A). In two clones, however, the number of detected species at harvest was lower than on the preceding day. This likely reflects technical limitations of LC–MS/MS detection at high total protein concentrations rather than a biological decrease in secreted HCPs. Across the pre-harvest time course, the product B clone (Clone 5) generally exhibited higher total HCP species counts than the product A clones (Clones 2 and 4) (Figure 1A). At the final harvest point, however, Clones 2 and 4 showed a pronounced increase in the number of detected HCP species, temporarily exceeding the levels observed in Clone 5. This late spike likely reflects process- or sampling-related factors like high cell lysis, influencing HCP release or MS detectability rather than a biological shift unique to product A.

By intersecting datasets across all clones, products, and processes, we identified a set of 351 proteins (28% of all HCP species) present in every sample (Figure 1B). Quantitative filtering for consistently measurable species yielded 67 proteins (the “quantifiable core”), representing only 5% of identified species. Notably, these 67 core proteins account for ∼63% of the total extracellular protein mass from cell culture supernatants. This disproportionate contribution illustrates that a small subset of highly abundant proteins accounts for the majority of the total HCP mass and therefore represents a major component of the cellular energy investment. Although these proteins dominate the mass balance, our data at this stage do not establish whether they contribute disproportionately to downstream purification burden. From a host cell engineering perspective, this is encouraging, as targeted removal of only a few key proteins could substantially reduce overall HCP levels while potentially liberating cellular capacity for product formation.

To assess the cellular cost of producing these proteins, we estimated translational and secretion energy requirements following previous publications (Kol et al., 2020). This allowed ranking of the core proteins by their predicted energetic cost in combination with their detected extracellular abundance from LC–MS/MS analysis. Since the proteomic dataset reflects only proteins present in the culture supernatant, it may underestimate proteins with high synthesis rates but rapid turnover or low release rate. To relate transcript-derived estimates to protein-level abundance, we compared RNA-seq expression values (transcripts per million, TPM) with mass-spectrometry–based HCP abundance (Figure 1C). TPM is used here as a normalized measure of transcript abundance. The energetic “cost” term was applied following published formulations but does not influence the relative relationship between the two datasets; rather, it serves to place transcript-derived and protein-derived metrics on a comparable conceptual scale. As expected, TPM and MS-based abundance showed a moderate positive correlation (Pearson r = 0.40, *R* ^2^ = 0.16, p < 0.001), indicating that transcript levels partially reflect extracellular HCP abundance but do not fully predict it. The majority of quantifiable core HCPs were low in abundance or energetic demand, whereas a smaller subset combined high energetic cost with high abundance. Within this group, thrombospondin-1 (THBS1) and fibronectin-1 (FN1) were among the most abundant and energy-intensive species, while EEF1A1 showed high transcript levels despite lower extracellular abundance.

To identify difficult-to-remove species, we analyzed HCPs that persisted across multiple DSP steps. Such targets are of particular interest, as their removal could simplify purification and increase drug safety. Four production clones (C1–C4) expressing the same IgG1 under matched fed-batch conditions were sampled for LC–MS/MS peptide mapping at harvest and after each major DSP step: Protein A capture (ProtA), virus inactivation (VI), cation exchange (CEX), and anion exchange (AEX) (Table 2).

**TABLE 2:** Summary of purification efficiency for HCP removal during downstream processing. The table lists the number of distinct HCP species detected at each purification step, the total and percentage reduction relative to harvest, and the remaining HCP concentration expressed in ng/mL, ppm, and as a percentage of total protein. Values represent means from four production clones.

Sample	Number of HCP species	Total Reduction [∼fold]	Reduction [%]	Amount of HCP [ppm]	Amount of HCP [ng/mL]
Harvest	1015	N/A	N/A	224,647	1,206,855.0
ProtA	50	311	99.68	736	3,565.0
VI	26	1549	79.95	149	715.0
CEX	12	3503	55.79	65	316.0
AEX	3	27013	87.03	8	41.0

Across all four clones, HCP removal exceeded 99.68% after ProtA chromatography and continued to improve with each subsequent DSP step, culminating in a ∼27,000-fold reduction after AEX (Figure 1D). The number of unique HCP species dropped from 1,015 at harvest to 50 after ProtA, 26 after VI, 12 after CEX, and just 3 after AEX (Figure 1E). Importantly, the individual DSP steps differed in the extent to which they reduced HCP levels. For example, the CEX step removed ∼56% of the remaining HCP mass and ∼54% of the remaining HCP species (Figure 1F; Table 2). These values reflect the position of CEX within the purification sequence rather than its intrinsic selectivity, and they should be interpreted in the context of the entire multimodal process, which removes additional impurities beyond HCPs. Despite these high removal efficiencies, several proteins consistently co-purified with the product across all clones at every DSP stage. Venn analyses were performed to define “core” HCPs, i.e., proteins detected in all four clones at a given purification stage. After ProtA, 23 core proteins remained, dominated by thrombospondin-1 (THBS1), which represented the largest fraction of residual HCP mass (Supplementary Figure S2A). Following VI, the core pool was reduced to 12 proteins, with THBS1 still accounting for ∼18% of the mass (Supplementary Figure S2B). CEX further narrowed the persistent HCP set to just three species: clusterin (CLU), peroxiredoxin-1 (PRDX1), and cathepsin B (CTSB), with CLU and PRDX1 together comprising two-thirds of the remaining HCP mass (Supplementary Figure S2C). After AEX, only two core proteins, Peroxiredoxin-1 (PRDX1) and Ubiquitin (UBC), remained, with PRDX1 contributing ∼41% of the residual HCP mass (Supplementary Figure S2D). Several HCPs remained detectable even after multiple orthogonal DSP steps, indicating product association or physicochemical properties conferring resistance to purification. These species represent key targets for genetic removal to further reduce residual HCPs early in processing.

For knockout screening, we pursued an unbiased yet structured target-selection strategy, considering extracellular abundance, predicted energetic cost, and persistence through DSP, while also including canonical lethal or fitness-associated genes to explore their potential dispensability in CHO cells. Candidates were retained or excluded based on (i) consistent detection across time and process conditions, (ii) abundance within the top 5%–10% of released HCPs, (iii) predicted secretion cost, and (iv) persistence potential inferred from DSP profiling. Targets failing to meet these criteria across batches were removed from subsequent engineering rounds. Using this funnel, we identified 67 abundant core HCPs, many of which overlapped with difficult-to-remove species.

In addition, we included four difficult-to-remove HCPs that were identified as part of the common set within each individual DSP step across all clones (Supplementary Figure S2). resulting in a total of 71 gene targets (Supplementary Table S1) taken forward into our evaluation pipeline.

### Engineering a cleaner CHO: systematic knockout screening of abundant and difficult-to-remove HCPs

**FIGURE 2:**
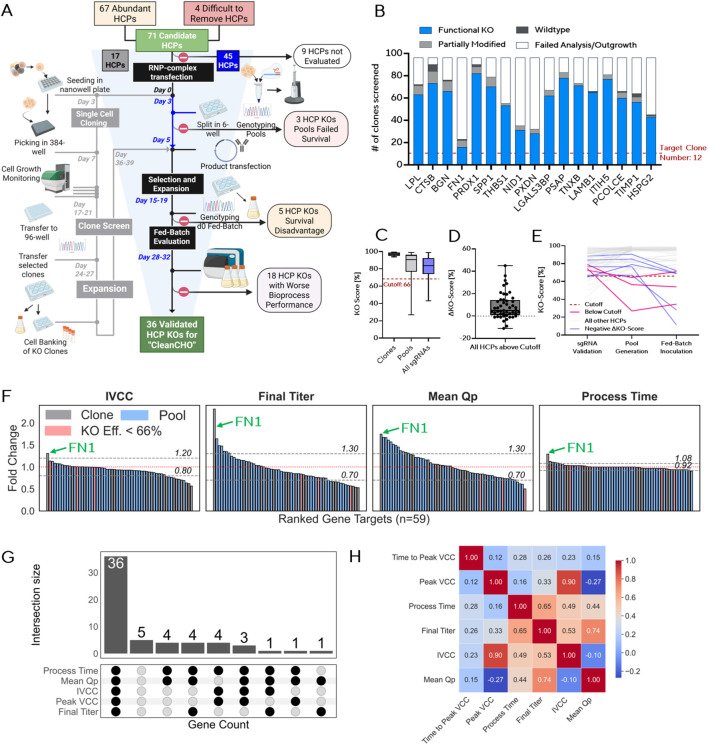
Systematic Genetic and Phenotypic Evaluation of HCP Knockouts. **(A)** Overview of the knockout screening pipeline. Of 71 prioritized HCPs, 59 transfected pools survived CRISPR RNP transfection, followed by single-cell cloning or pooled evaluation. Editing validation, expansion, and fed-batch evaluation were performed for both clones and high-efficiency KO pools. 36 HCP knockouts were retained for the CleanCHO panel based on bioprocess neutrality. **(B)** Editing spectrum of 96 single-cell clones from 17 HCP knockouts. Stacked bars indicate editing outcomes: Functional KO, partially modified, wildtype and failed analysis. **(C)** Distribution of knockout scores in all generated clones and final pools, additionally the KO scores for the top sgRNA per gene during sgRNA screening is shown. Red dotted line: 66% KO efficiency cutoff for stable pool generation (across all pool evaluations). **(D)** Increases in KO Scores from multiple transfections with the sgRNA Validation as a starting point across all pool evaluations. **(E)** Longitudinal KO score tracking across transfection timepoints and fed batch inoculation for all evaluated HCP targets. Targets falling below KO score thresholds are marked (66%). **(F)** Fold change relative to wild-type control for four key bioprocess variables: IVCC, final titer, mean Qp, and process duration. Gray bars: clone-based runs. Blue bars: pool-based runs. Gray dashed lines: WT cutoff bounds (approximately ∼ 2-times SD). Red dashed lines: WT mean. **(G)** UpSet plot showing overlaps of performance-impacting knockouts across the four evaluated variables. Most knockouts did not display detrimental effects across multiple metrics. Knockouts with declining KO scores were excluded from process criteria, as no reliable conclusions could be drawn. Dots indicate matching wild-type process performance, while bars show the number of genes in each group. **(H)** Pearson correlation heatmap of all evaluated bioprocess parameters. Strong correlations are observed between IVCC, Process Time and Final Titer. Figure 2A: Created in BioRender. Marzluf, J. (2026). https://BioRender.com/uc0wre4.

To determine the suitability of individual HCP knockouts for the development of a cleaner CHO cell line, we implemented a multistep evaluation workflow combining genetic validation, bioprocess evaluation and scoring against wild-type benchmarks (Figure 2A). From the 71 candidate HCPs identified in our abundance and difficult-to-remove screen, 62 were subjected to CRISPR-based KO. The missing 9 targets were not evaluated due to design constrictions and or failure to establish PCR reactions for genotyping. 17 targets were evaluated in clonal format, while 45 were tested in stable KO pools. For the clonal arm, 1,632 single-cell clones were generated across the 17 genes in five screening runs, yielding 96 clones per gene for genetic characterization, of which ∼12 clones were expanded and evaluated in fed-batch experiments. For the pooled arm, 135 stable KO pools across 45 HCPs were generated in seven runs. Pools were established by sequential CRISPR RNP transfections and genotyped. Multiple transfections increased knockout efficiencies relative to single transfections (Marzluf et al., 2025b; Carver et al., 2022). All runs included an unedited wild-type pool as control and to correct for batch effects. KO pools were re-genotyped at fed-batch inoculation, and targets with unstable KO scores (absolute change >10%) were excluded. An overview summarizing all results is shown in Figure 2A.

Genetic characterization of the clonal knockouts confirmed a high fraction of functional KOs, with almost all generated clones harboring complete loss of function mutations (Figure 2B). Only a small subset retained wild-type alleles or in-frame edits. For each gene, clones with verified disruptive genotypes and normal growth morphology were prioritized, while those with ambiguous PCR results or poor growth were excluded. On average, twelve validated KO clones per gene were selected for expansion, product transfection, and fed-batch evaluation.

For the pooled KO screening approach, we optimized editing outcomes by screening three sgRNAs per target (Figure 2C). We defined a minimum KO score of 66% for KO pool generation prior to product transfection and fed-batch inoculation. Sequential transfections improved KO scores for most sgRNAs, with a median gain of 5% and maximum increases of up to 45% compared to initial sgRNA validation (Figure 2D). In some targets, however, KO scores declined between initial validation, pool generation, and fed-batch inoculation, indicating partial loss of edited alleles over time (Figure 2E). Such KO-score drift likely reflects selective pressures during pool expansion, including outgrowth of partially edited subpopulations, or gene KO related fitness effects. However, most sgRNAs already exhibited high efficiencies, with the best-performing sgRNA per gene reaching a median KO score of 83.5% and a mean of 81.9%. The difference between pool validation and fed-batch inoculation served as an additional exclusion criterion, as marked score declines suggested overgrowth by incompletely edited subpopulations (Figure 2E).

The rationale for pooled KO screening was based on our previously published methodology (Marzluf et al., 2025b): While single-cell cloning enables precise genetic assessment, it extended the evaluation timeline by 4–6 weeks and introduced additional variability between clones, as reflected by higher variance across bioprocess performance metrics such as titer, IVCC, and Qp (Supplementary Figure S3). By contrast, pooled knockouts offered several advantages: (i) most clones obtained after single transfection already carried functional KOs (Figure 2B) (across 17 genes a mean of 90.0% clones with an outcome harbored a full functional KO), rendering cloning largely redundant, (ii) clonal variability often obscured phenotypic effects, while pools better represent the singular effect of the knockout, whereas individual clones often introduce additional variability unrelated to the targeted edit. The validity of this pooled screening approach was further supported by consistent genotype–phenotype correlations observed for the model target FUT8 and the top hit FN1 in a previous study (Marzluf et al., 2025b). Based on these observations, pooled screening was prioritized for most HCP KO candidates (45 genes).

Of the 71 HCP candidates initially identified, 59 yielded viable knockout populations and were subsequently evaluated for phenotypic impact. Each KO clone or pool was transfected with the same IgG1 expression plasmid and stable producer cell lines were generated. In total, five KO clone runs (Supplementary Figure S4) and seven KO pool runs (Supplementary Figures S5–S6) were conducted and analyzed.

For statistical evaluation, we focused on four key bioprocess parameters: integral viable cell concentration (IVCC), final titer, mean cell-specific productivity (Qp), and process duration. Across these controls, intra-batch WT variance was generally low (Supplementary Figure S7A): Process parameters Peak VCC, Process Time and IVCC showed moderate ∼5–15% variation while the highest variation observed in final titer and Mean Qp was at ∼30%. To identify deviations beyond wild-type variation, we defined cutoff thresholds based on the observed standard deviation, applying approximately two-fold margins taking into account biological variation (Supplementary Figure S7A). The tolerance was slightly relaxed for each parameter, acknowledging that some HCP knockouts, particularly those difficult to remove during DSP, could be beneficial even if associated with modest reductions in bioprocess performance. KO clones or pools falling below the cutoff were classified as negative hits, while those exceeding it were considered promising candidates for further validation in multiplexed setups. Bar plot rankings of fold change across all evaluated knockouts confirmed a largely neutral phenotype for most candidates (Figure 2F). Of 59 KO targets screened, 36 passed the lower performance threshold for four set performance criteria and were retained as hits qualified for further investigation in multiplex KO approaches (Figure 2G). A small subset of KOs (e.g., PRDX1, CTSB, HSPA5) showed reduced growth or productivity (Supplementary Figure S4), while FN1 exhibited performance improvements in final titer (∼2-fold increase), IVCC (∼1.3-fold increase) and process time (∼1.2 fold increase) (Figure 2F) (Supplementary Figure S4).

Correlation analysis between process metrics confirmed expected interdependencies, including a strong correlation between peak VCC and IVCC (Pearson r = 0.90) and a high correlation between process duration and final titer (Pearson r = 0.65) (Figure 2H) (Supplementary Figure S7B). Final titer correlated moderately with IVCC (Pearson r = 0.53) and weaker with peak VCC (Pearson r = 0.33). Notably, peak VCC showed a weak negative correlation with Qp (Pearson r = −0.27). This aligns with previous reports describing an inverse relationship between growth and productivity in CHO fed-batch processes (Bryan et al., 2021; Altamirano et al., 2001), reflecting the trade-off between biomass accumulation and recombinant protein secretion.

These findings underscore the potential for broad HCP removal without compromising upstream process integrity. Summarizing the total HCP mass from our discovery dataset for all validated HCPs, total HCP content may be reduced by 32%.

### Fibronectin-1 KO consistently improves bioprocess performance

**FIGURE 3:**
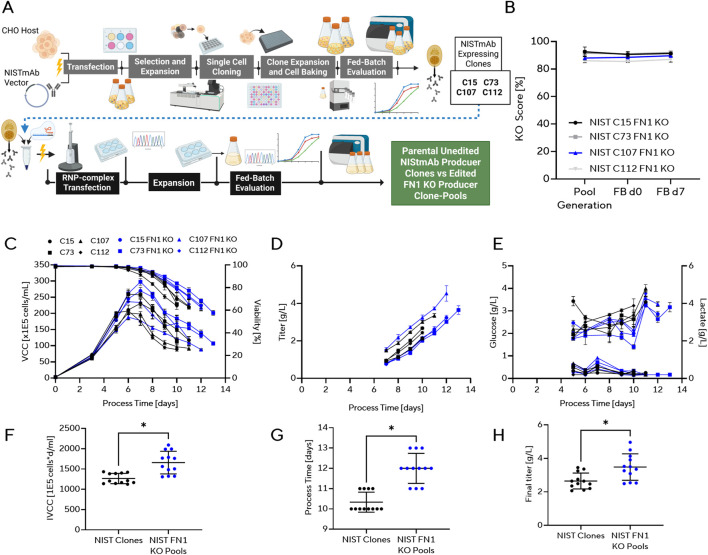
Re-Validation of FN1 KO in NISTmAb-producing CHO clones. **(A)** Schematic of the experimental workflow for generating and evaluating FN1 KO producer clones and pools. Parental NISTmAb-expressing CHO clones (C15, C73, C107, C112) were compared with corresponding FN1 KO derivative pools. NISTmAb expression clones were generated by classical cell line development. These clones were then edited by transfection, generating clone-based KO-Pools, followed by expansion and fed-batch characterization. **(B)** KO scores for FN1-edited NIST clone-pools measured during pool generation, fed-batch inoculation (d0), and Day 7 of the fed-batch (d7). (n = 3) **(C–E)** Fed-batch performance of parental and FN1 KO clones and pools: **(C)** VCC and viability, **(D)** titer, and **(E)** glucose and lactate concentrations across a 14-day process. Data represent technical triplicates (n = 3) with mean ± standard deviation across all four clones. **(F–H)** Endpoint process metrics for NIST parental clones compared with FN1 KO pools: **(F)** IVCC, **(G)** process duration, and **(H)** final titer. Data represents all measurements in technical replicates (n = 3) across all clones for both groups (n = 4 biological replicates) presented as mean ± standard deviation. Statistical significance was assessed by unpaired two-tailed t-test; *p < 0.05. Figure 3A: Created in BioRender. Marzluf, J. (2026). https://BioRender.com/9lmbq29.

To further validate the phenotypic impact of FN1 knockout, identified as a top hit in the screening of HCP KO candidates, we introduced FN1 KOs into four established NISTmAb (National Institute of Standards and Technology NIST, 2016) producer clones and evaluated their performance in fed-batch culture (Figure 3A). Consistent with the pooled screening platform used in this study and our previous work (Marzluf et al., 2025b), FN1 knockouts were implemented as stable KO pools derived from each NISTmAb clone. NISTmAb producer clones were transfected with a validated sgRNA targeting an early FN1 exon. KO scores remained stable across pool generation, fed-batch inoculation, and peak cell growth of the bioprocess, maintaining ∼90% editing efficiency (Figure 3B). In fed-batch experiments, FN1 KO pools derived from each parental clone displayed extended stationary phases and delayed onset of viability decline compared with controls (Figure 3C). This phenotype was consistently associated with prolonged culture duration and higher IVCC. As a consequence, FN1 KO pools achieved significantly increased final titers, with average improvements exceeding ∼30% relative to parental clones (Figures 3D,H). Glucose consumption and lactate accumulation were unchanged between FN1 KO and parental clones, indicating that the improvements were not driven by altered central carbon metabolism (Figure 3E). Instead, the phenotype reflects enhanced culture longevity, as evidenced by higher IVCC and extended process time (Figures 3F,G). Together, these results confirm that FN1 knockout delays the onset of cell death, extends effective production time, and increases cumulative product yield. Importantly, this beneficial effect was reproduced across four independent producer clones and aligns with previous findings (Marzluf et al., 2025b), supporting FN1 KO as a broadly applicable strategy to enhance CHO production cell line performance.

### Moderate decreases in released HCPs via 7-fold multiplex knockouts

**FIGURE 4:**
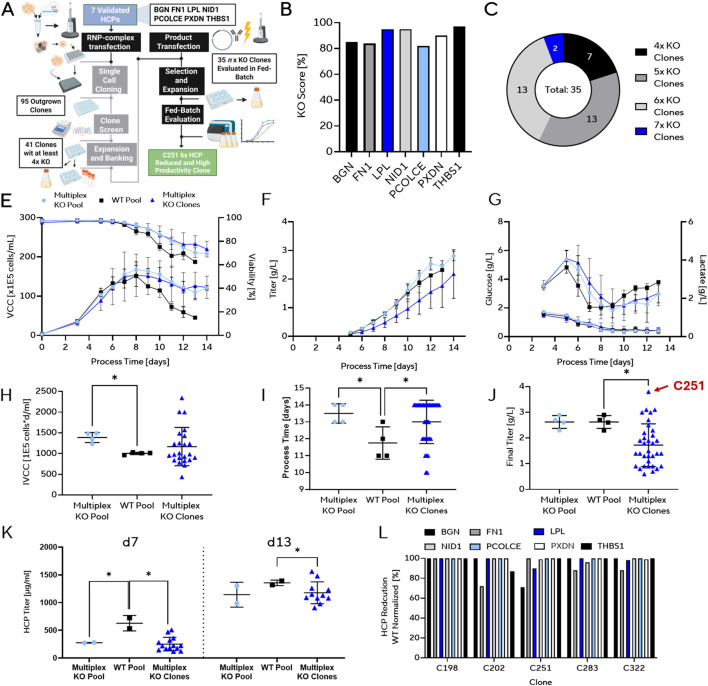
Multiplex genome editing enables combined knockout of seven HCPs. **(A)** Workflow for multiplex knockout generation and evaluation. The 7× multiplex KO pool was generated by RNP-complex transfection targeting BGN, FN1, LPL, NID1, PCOLCE, PXDN and THBS1, followed by single-cell cloning, expansion, and fed-batch evaluation. In total, 95 clones were screened. **(B,C)** Genetic characterization of multiplex KO pool and clones. **(B)** Bar plot showing editing efficiency in the generated KO pool, and **(C)** distribution of multiplex genotypes with 4×, 5×, 6×, and 7× knockout constellations recovered. **(D–F)** Fed-batch profiles of viable cell concentration (VCC) and viability **(D)**, as well as titer **(E)** and Glucose and Lactate levels **(F)** comparing parental 7x multiplex KO pool clones with wild-type (WT) controls. Data represent mean values ±standard deviation of all replicates in each group (n = 4 technical replicates for Multiplex KO Pools and WT Pool; n = 35 KO clones) **(G–I)** Endpoint process parameters for 7× multiplex KO pool and clones compared with WT: **(G)** integral viable cell concentration (IVCC), **(H)** process duration, and **(I)** final titer. **(J)** HCP reduction in 7× KO Pool and derived clones compared with WT, showing consistently reduced levels across multiple replicates. Data represent mean values ±standard deviation of all replicates in each group (n = 4 technical replicates for Multiplex KO Pools and WT Pool; n = 35 KO clones) **(K)** Validation of a selection of multiplex KO clones by LC–MS/MS confirming loss of target proteins, in the 7× multiplex KO pool background. Figure 4A: Created in BioRender. Marzluf, J. (2026). https://BioRender.com/9fesdm0.

To validate to what degree multiplexed HCP KOs can lower overall HCP content, we aimed to generate seven-fold knockout (7× KO) cell lines targeting BGN, FN1, LPL, NID1, PCOLCE, PXDN and THBS1 (Figure 4A). Together, these seven proteins accounted for ∼15% of the extracellular HCP mass in our initial discovery dataset. After transfection, the 7× KO pool showed high editing efficiency, with all HCP genes showing KO scores above 80.0%. High editing efficiency is crucial during pool generation for the probabilistic recovery of full 7× multiplex KO clones. This edited pool was subjected to single-cell cloning, yielding 95 outgrown clones for genotyping. Genetic analysis revealed a range of multiplex HCP KO configurations: two full 7× KOs, with the remaining clones harboring 6× KOs, 5× KOs (37% each) and 4× KOs (20%) (Figures 4B,C). These clones were grouped for downstream analysis because no consistent genotype-specific trends in growth, productivity, or HCP reduction were observed across individual KO combinations, and phenotypic variability was dominated by clonal background rather than by specific target composition or KO number within the ≥4× KO group. All clones with sub-combinations of at least 4× KOs are referred to as multiplex KO clones going forward. These 35 clones were expanded and transfected with a standard IgG1 expression vector to create stable producer pools. After selection and expansion, these cell lines were subjected to fed-batch evaluation for comparative assessment of growth, viability, productivity, and HCP levels.

Fed-batch evaluation revealed that both the 7× KO pool and derived multiplex KO clones exhibited a markedly prolonged stationary phase compared with wild-type (WT) controls (Figure 4D). No changes in Glucose consumption or lactate secretion are observed (Figure 4F). This phenotype is likely driven by the KO of FN1, that is consistently present in most multiplex KO clones. This shift in culture longevity translated into increased IVCC and extended process duration relative to WT (Figures 4G,H). Final titers of multiplex KO pools were comparable to WT, whereas clone-derived KOs displayed greater variability, with some clones yielding reduced titers (Figures 4E,I). Although average titers were lower in the multiplex KO clones, we demonstrate that highly productive clones can be generated from the 7× KO pool with titers exceeding those observed in the parental pool or the WT control. Analysis of bulk HCP levels confirmed that multiplex KO pools and clones achieved large reductions in total HCP content. On day 7 of the fed-batch, secreted HCP content was reduced by ∼58% in both 7× KO pools (275.5 μg/mL) and clone-derived KOs (251.9 μg/mL) compared with WT controls (628.0 μg/mL) (Figure 4J). Based on our initial HCP dataset, the targeted seven proteins together account for ∼15% of total HCP mass. However, the observed reduction was nearly fourfold higher suggesting that multiplex genome editing, even in partially edited clones, suppressed HCP secretion beyond the direct loss of targeted proteins. This effect is particularly notable given that most clones did not harbor complete 7× KO genotypes. Since clonal variation naturally introduces differences in HCP expression, further optimization through careful clone selection is likely to yield additional reductions. At later process stages (day 13), the apparent HCP reduction diminished, likely because increased cell lysis released intracellular proteins into the supernatant, thereby masking the earlier benefit of reduced secreted HCPs (Park et al., 2017). At this late stage, the HCP content was more in line with our prediction, HCP content was reduced by ∼15% in both 7× KO pools (1142.0 μg/mL) and clone-derived KOs (1176.8 μg/mL) compared with WT controls (1355.0 μg/mL) (Figure 4J).

To validate HCP loss at the targeted protein level, we used MS peptide mapping, which offers high sensitivity for detecting individual host proteins. We selected clones covering the full range of HCP secretion and productivity phenotypes: C198 (6× KO, high HCP, 0.7 g/L mAb), C202 (7× KO, low HCP, 1.3 g/L mAb), C251 (6× KO, low HCP, 3.8 g/L mAb, top producer), C283 (7× KO, average HCP, 2.2 g/L mAb), and C322 (7× KO, high HCP, 3.0 g/L mAb), alongside the wild-type control (WT1, high HCP, 2.1 g/L mAb), as determined by bulk HCP quantification with Octet ELISA. MS-protein-level analysis confirmed near-complete elimination of the seven targeted HCPs in most multiplex KO clones (Figure 4K). However, residual expression of BGN (≤30% of WT) in clone C251 reflects incomplete gene disruption for the BGN gene, as the clone retained one unmodified allele or in-frame coding mutation, leading to residual translated output.

Taken together, the multiplex KOs reduced secretion of targeted HCPs, though residual expression persisted in some clones to a minimal level due to incomplete editing and/or escape mechanisms. Further reductions may be achieved through additional rounds of engineering and by carefully selecting clones with minimal residual HCP production. Based on its high productivity, clone C251 was advanced for a subsequent KO round.

### Enhanced decreases in released HCPs via 11-fold multiplex knockouts

**FIGURE 5:**
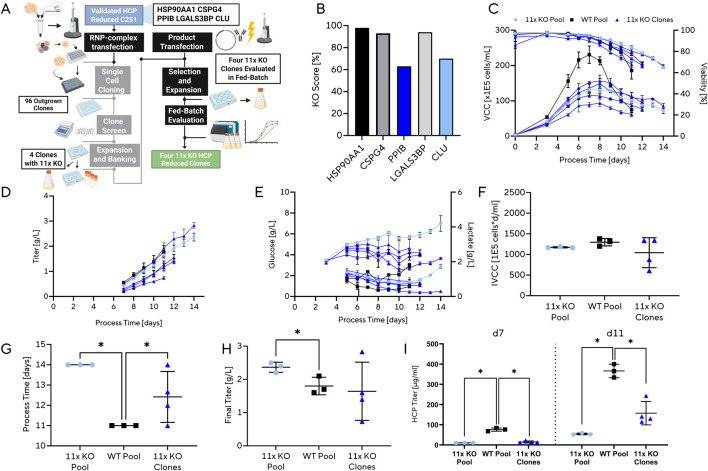
Extension of multiplex genome editing to 11 host cell protein (HCP) knockouts in CHO. **(A)** Workflow for generation and evaluation of extended multiplex KO clones. Starting from a previously established 6× KO background, five additional HCP targets (HSP90AA1, CSPG4, PPIB, LGALS3BP, CLU) were disrupted by RNP-complex transfection. Single-cell clones were expanded, banked, and evaluated in fed-batch culture. In total, 96 clones were screened, and 4 clones harboring all 11 intended knockouts were recovered, of which 4 were further analyzed in fed-batch. **(B)** Editing efficiency of generated KO pool (n = 1). **(C–E)** Fed-batch performance of 11× KO clones compared with WT background: **(C)** viable cell concentration (VCC) and viability, **(D)** titer, and **(E)** glucose and lactate concentrations. Each KO clone is plotted as the mean of three technical replicates; the 11× KO pool and WT pool are plotted from three biological replicates each. Data are shown as mean ± standard deviation. **(F–H)** Endpoint process parameters of 11× KO clones compared with WT controls: **(F)** integral viable cell concentration (IVCC), **(G)** process duration, and **(H)** final titer. Data are shown as mean ± standard deviation. **(I)** HCP reduction in 11x KO pool and derived clones compared with WT, showing consistently reduced levels across multiple replicates. Each KO clone is plotted as the mean of three technical replicates; the 11× KO pool and WT pool are plotted from three biological replicates each. Data are shown as mean ± standard deviation. Figure 5A: Created in BioRender. Marzluf, J. (2026). https://BioRender.com/zv7fda7.

To further expand the multiplex HCP knockout strategy, we selected clone C251, which had previously been identified as both highly productive and exhibiting a moderate reduction in HCP secretion in the previous experiment, as the starting point for additional engineering. Clone C251 showed partial KO for BGN and full functional KO for FN1, LPL, NID1, PCOLCE, PXDN and THBS1 defining it as the 6x parental KO Clone C251. Five additional HCPs (HSP90AA1, CSPG4, PPIB, LGALS3BP, and CLU) were targeted using CRISPR RNP transfection, generating an 11× KO pool (Figure 5A). Editing efficiency was high (>80%) for three of the genes, while PPIB and CLU showed lower efficiencies, only exceeding ∼50% (Figure 5B). From 96 single-cell clones screened, four were confirmed to harbor complete 11-fold knockouts, consistent with the lower recovery rate expected due to the reduced editing efficiency at two of the target loci. These clones were subsequently expanded, banked, and evaluated in fed-batch culture following transfection with a standard IgG1 expression construct.

Fed-batch evaluation of the four 11× KO clones and their parental C251-derived 11× KO pool revealed reduced cell growth consistent with the previous experiment, characterized by decreased peak VCC, while still maintaining an FN1-like phenotype with extended stationary phase and delayed onset of viability decline (Figure 5C). Titer profiles showed substantial variability across the four KO clones: three exhibited markedly lower titer accumulation, whereas one high-performing clone, together with the 11× KO pool, achieved elevated final titers compared with the WT control (Figures 5D,H). Glucose and lactate measurements indicated strongly reduced glucose consumption in both the 11× KO pool and its derived clones, likely reflecting the decreased cell growth and biomass accumulation (Figure 5E). IVCC values were comparable between the WT pool, 11× KO pool and its derived clones (Figure 5F). In addition to lower peak VCC, process duration was extended in the 11× KO pool and its clones (Figure 5G), enabling increased cumulative production. This variability likely reflects clonal background heterogeneity introduced during single-cell cloning rather than uniform editing-related effects, as all clones share the same 11× knockout genotype but differ in chromosomal context and epigenetic state. This highlights the need to generate and evaluate additional 11× KO clones to capture the full range of phenotypic variation within the parental pool and to identify stable, high-performing candidates. As previously reported, the inherent variability among CHO subclones necessitates large sample sizes for statistically robust conclusions: detecting a 0.3-fold difference in final titer with 90% power would require ∼141 individual clones, while ∼10–20 may suffice at 80% power (Marzluf et al., 2025b). In this study, only four 11× KO clones were analyzed, yet one already showed superior performance. This clone is therefore presented as a proof-of-feasibility example rather than as evidence of a deterministic, genotype-driven productivity improvement. For development of a new production host, expanding clonal recovery and evaluation would therefore be critical to confirm that observed improvements are consistent and reproducible. Particularly notable was the successful inclusion of CLU in the knockout panel, since clusterin belongs to the class of difficult-to-remove HCPs.

Analysis of HCP levels revealed further reductions beyond those observed in the 7× KO pool and clones. On day 11, HCP content was reduced by ∼85% in 11× KO pools (54.6 μg/mL) and by ∼57% in clone-derived KOs (157.4 μg/mL) compared with WT controls (366.2 μg/mL) (Figure 5I). This striking difference between pools and clones may be partially explained by lower biomass accumulation in the KO pools, which would reduce the total HCP burden. Relative to the 6× KO parental C251 clone derived from our target 7× KO run, the 11× KO clones showed an additional ∼20% reduction in HCP content. By day 11, these differences were less pronounced, consistent with increasing contributions of intracellular proteins released during late-stage culture cell lysis, a phenomenon also observed in the 7× KO analysis.

Together, these data demonstrate that iterative multiplex editing is feasible in CHO cells and enables stepwise elimination of an increasing number of secreted HCPs without detrimental effects on growth or productivity, while further lowering total HCP levels beyond initial knockout rounds.

## Discussion

A small fraction of extracellular proteins contribute to the majority of total HCP mass, and several species persist through orthogonal purification steps (Kol et al., 2020; Valente et al., 2018; Park et al., 2017; Valente et al., 2015). Studies have repeatedly identified adhesive ECM proteins (e.g., THBS1, FN1) and chaperones/cofactors (e.g., CLU, PRDX1) as recurrent co-purifiers after Protein A and ion-exchange purification steps, likely owing to product association, multimeric state, or physicochemical similarity to IgG (Chiu et al., 2017; Valente et al., 2015). In our study, we identified abundant and difficult-to-remove HCPs for targeted genetic engineering of CHO cells to reduce HCP burden. About ∼60% of HCP species appeared well before loss of viability, which is consistent with reports that secretion drives HCP release early to mid-process while cell lysis contributes mainly near harvest (Bracewell et al., 2015). Clonal background and product identity both shaped the HCP composition: clones from the same transfection pool showed higher similarity than unrelated clones, and identical mAb products shared a greater fraction of HCPs compared with cross-product comparisons. These trends are consistent with evidence that the CHO secretome reflects both lineage-specific traits (Hamaker et al., 2022), and transgene/product properties (Park et al., 2017). Despite process-dependent variation, a stable core set of HCPs was observed, and only 67 proteins accounted for ∼63% of total extracellular mass, highlighting that a small subset of highly abundant species drives much of the metabolic and downstream burden.

The generation of HCP KO CHO lines represents a promising strategy toward creating a “clean” production host with reduced downstream purification burden and potentially alleviated metabolism. Across multiple KO screen runs, candidate HCP-genes were assessed based on their effects on growth, viability and productivity in fed-batch processes. Several HCPs were found to be essential for cell survival or hindered proliferation, and their disruption impaired cell viability. Pools generated with KOs of GAPDH, ALDOA, and EEF2 failed to show recovery after transfections, in line with their central roles in glycolysis and protein synthesis (Phadke et al., 2011; Moore et al., 2016; Ji et al., 2016). Similarly, five additional HCP KO pools (EEF1A1, PGK1, LDHA, TINAGL1, and NME1) proved unstable, reverting toward wild-type alleles by the time of fed-batch inoculation, suggesting strong selective disadvantage of those disruptions. More generally, longitudinal changes in KO scores across validation, pool generation, and fed-batch inoculation likely reflect a combination of fitness-associated selection and allele-level escape mechanisms. Complete loss of certain loci may impose subtle growth or stress disadvantages, enabling partially edited subclones or cells carrying in-frame indels to outcompete full knockouts during expansion. In pooled CRISPR-edited populations, such effects can be amplified over extended culture periods, even when initial editing efficiencies are high (Smits et al., 2019). These observations underscore the importance of longitudinal KO-score monitoring and conservative exclusion criteria when interpreting pooled or multiplex knockout phenotypes in CHO. For example, knockdown or disruption of glycolytic enzymes such as LDHA has been shown to perturb cell metabolism and viability in CHO systems (Kalkan et al., 2023). EEF1A1 is a core translation elongation factor whose perturbation impairs proliferation in multiple cell models (Amiri et al., 2023; Zhang et al., 2024; Liu et al., 2024). PGK1 is similarly central in glycolysis and often intolerant to loss-of-function editing (Jin et al., 2020; Pereira et al., 2018). Finally, PRDX1 disrupted single cell clones, while functional, showed impaired growth and productivity confirming its importance in stress response and oxidative balance (Han et al., 2016; Moriwaki et al., 2021). This is especially unfortunate for a protein like PRDX1 since it is one of the major persistent proteins during downstream processing but cannot be removed by genetic disruption according to our results. Additionally, the previously published CHO_EG2025 essential gene set identified EEF1A1, PGK1, LDHA, and NME1 as required for cell proliferation or as fitness genes, as their knockout resulted in clearance in pooled CRISPR screens (Marzluf et al., 2025a).

Some knockouts, while not lethal, impaired bioprocess parameters. ACTB and PPIA disruption reduced peak viable cell concentrations without strongly affecting productivity, but potential risks to protein quality were noted due to their roles in cytoskeletal organization and protein folding (Nigro et al., 2013; Vanslembrouck et al., 2020). KO of HSP90B1 decreased growth and titers, consistent with its role in protein folding and ER homeostasis (Jossé et al., 2012). HTRA1 and YWHAZ disruption lowered final titers, indicating impaired protein stability or secretory network imbalance (Eigenbrot et al., 2012; Sun and Lei, 2023). These targets are not recommended for multiplexing. These proteins are therefore unsuitable for the development of a “clean” CHO.

A large number of HCP knockouts showed no detrimental effects on growth or productivity, making them strong candidates for multiplexing. This group includes most of the 12 genes targeted for our multiplexing KO study: LPL, BGN, FN1, THBS-1, NID1, PCOLCE, PXDN, HSP90AA1, CSPG4, PPIB, LGALS3BP and CLU, all of which performed comparably to WT pools, confirming previous reports for BGN and LPL (Kol et al., 2020; Chiu et al., 2017). Similarly, CTSA disruption had no adverse effects, with its KO being particularly promising due to its role in product fragmentation during downstream processing (Dovgan et al., 2021).

A small subset of KO targets not only had no negative effects but also improved process performance. FN1 disruption consistently enhanced culture longevity and titers, likely by reducing aggregation and apoptosis through loss of extracellular matrix interactions (Renner et al., 1993; Klingler et al., 2021). This was validated in both clone- and pool-based systems in a previous study (Marzluf et al., 2025b). Similarly, CTSB KO clones displayed prolonged stationary phases and high productivity, despite slightly impaired growth (Sahoo et al., 2024). CLU also showed increased productivity and prolonged high viability (Wilson and Easterbrook-Smith, 2000).

To further validate our top hit FN1, we introduced FN1 KOs into four distinct NISTmAb producer clones. Here, the edited cells consistently showed delayed loss of viability, extended process time, and increased final titer without marked changes in glucose/lactate profiles. FN1 is an abundant extracellular matrix protein frequently detected in HCP impurity maps (Valente et al., 2018; Klingler et al., 2021). Altered ECM signaling has been linked to extended culture longevity in CHO, providing a plausible mechanistic basis for the phenotype (Yamada and Even-Ram, 2002). The clone-agnostic reproduction across four producers aligns with prior reports of FN1-associated longevity effects in pooled formats (Marzluf et al., 2025b).

The 7-gene KO panel (BGN, FN1, LPL, NID1, PCOLCE, PXDN, THBS1) achieved ∼58% reduction in HCP by day 7, outpacing the ∼15% mass fraction of those specific proteins precited from our proteomic data. This disproportionate reduction suggests that multiplex HCP knockout induces secondary, system-level effects beyond the removal of targeted proteins. One plausible mechanism is that deletion of structural ECM components such as FN1 and THBS1 may disrupt co-secretion or retention of other extracellular matrix proteins, thereby reducing the overall HCP burden (Hynes, 2009). Additionally, eliminating highly abundant secreted proteins likely reduces the folding and trafficking load on the endoplasmic reticulum, which may in turn decrease secretion of other non-essential host proteins (Kol et al., 2020). The altered growth and viability kinetics observed in multiplex KO cultures, particularly the FN1-associated extension of the stationary phase, may also limit the release of lysis-derived HCPs during early to mid-process (Park et al., 2017). Together, these effects likely act in concert to amplify HCP reduction beyond the mass fraction of the directly targeted proteins.

Over time (by day 13), the gap in HCP mass narrows substantially, likely due to lysis-derived intracellular proteins contributing more to total HCP-mass (Bracewell et al., 2015). Mass spectrometry analysis confirmed the near-complete loss of the seven targets in most multiplex clones. However, one high producer showed residual levels of BGN/LPL which is in line with retained in-frame alleles or transcript-level escape (alternative splicing/exon skipping) that can maintain partial expression after frameshifts (Smits et al., 2019; Tuladhar et al., 2019; Sharpe and Cooper, 2017; Mou et al., 2017).

Iterative editing to yield 11× KOs in the highest performing 6× KO background (C251) was feasible, with reduced peak VCC but extended stationary phase, again reflecting a FN1-like phenotypic shift toward extended stationary phase and elevated viability. Titer outcomes diverged by clone, a pattern expected in CHO given chromosomal rearrangements, epigenetic divergence, and selection bottlenecks inherent to single-cell cloning (Vcelar et al., 2018; Vcelar et al., 2018; Pilbrough et al., 2009; Frye et al., 2016). Accordingly, the high-performing C251-derived 11× KO clone should not be interpreted as representative of all clones carrying this genotype, but rather as an illustration that high productivity can be retained within heavily edited backgrounds. The inclusion of CLU in the 11× multiplex KO is noteworthy: CLU is a recurrent, difficult-to-clear impurity after Protein A purification and polishing (Chiu et al., 2017; Valente et al., 2015). The stronger HCP reduction observed at day 11 in pools (∼85% vs. WT) compared with clones (∼57% vs. WT) likely reflects both clonal heterogeneity and the earlier loss of viability in three of the four recovered 11× KO clones. At this late stage, substantial lysis had likely contributed to the HCP background, whereas the 11× KO pool and the top-performing clone maintained extended viability. For developing new production host cell lines, this highlights the importance of screening a sufficiently large number of clones to recover those with favorable growth, viability, and productivity profiles. As shown here by second round clones representing the C251 decrease growth phenotype, and in other reports, sequential rounds of cloning and editing carry the risk of introducing an early selection bias, where the characteristics of an initially chosen clone can constrain the entire lineage of derived cell lines (Kol et al., 2020; Weiß et al., 2025; Carver et al., 2022). Marked variability in productivity among the 11× KO clones is consistent with the well-documented clonal heterogeneity of CHO cells. Importantly, no severe growth defects were detected, confirming that cumulative knockout of up to eleven HCPs did not compromise essential cellular functions under fed-batch conditions.

The data generated for this study support a dual cell-engineering strategy: (i) remove abundant and difficult-to-remove HCPs to reduce HCP load ahead of Protein A purification; and (ii) reduce the metabolic burden imposed by the expression of said HCPs. Additionally, we have identified FN1 as a beneficial KO to extend the fed-batch culture duration and simultaneously increase final titers. Attenuation of differences near harvest in HCP levels between engineered and WT cells argues for process-control strategies that terminate cultures earlier, at higher viabilities, while still maintaining high productivity. Several studies have demonstrated that adjusting feeding regimes, temperature shifts, or other process parameters can delay viability loss and reduce late-stage HCP release (Bracewell et al., 2015; Geng et al., 2024; Goey et al., 2017). Alternatively, genetic interventions that target apoptotic pathways, such as BAX/BAK knockouts, have been shown to suppress programmed cell death, prolong culture longevity, and lower the contribution of lysis-derived impurities in CHO bioprocesses (Tang et al., 2022; Lam et al., 2024; MacDonald et al., 2022).

Beyond upstream process performance, the development of a “clean” CHO chassis has direct translational relevance for biopharmaceutical manufacturing. Reducing the abundance and variability of host cell proteins at the source can improve robustness and consistency of downstream purification, potentially lowering reliance on extensive polishing steps and mitigating lot-to-lot variability. From a regulatory perspective, a simplified and more predictable HCP profile may facilitate impurity risk assessment and comparability evaluations during process changes or lifecycle management. Together, these aspects position clean-CHO host platforms as a complementary strategy to process optimization for improving manufacturing efficiency and product consistency. This study demonstrates that a small subset of highly abundant and difficult-to-remove HCPs is causing much of the metabolic and downstream processing burden in CHO. Systematic knockout screening distinguished essential from dispensable released proteins, identifying a panel of neutral or beneficial candidates for multiplex editing. Multiplex knockouts of up to eleven genes achieved substantial HCP reductions (∼57–85%), while FN1 disruption consistently improved culture longevity and titers. These results highlight two complementary engineering strategies: eliminating abundant/difficult-to-remove HCPs to reduce DSP load and metabolic cost, and selectively removing HCPs like FN1 to enhance productivity. Together, this approach offers an additional path toward a “clean” CHO production platform with lower impurity levels, extended culture performance improved manufacturing efficiency and ultimately safer drug for more patients.

## Concluding remarks

We show that a small set of abundant, DSP-persistent HCPs accounts for most extracellular burden, and that multiplex CRISPR gene editing can yield the removal (up to 11 genes) can cut HCPs by ∼57–85% without compromising core fitness. Beyond purity, the knockout of FN1 consistently extended culture longevity and raised titers, demonstrating that select HCP edits can improve both upstream performance and downstream simplicity. Late-stage convergence of HCPs highlights two levers: earlier harvest/viability-focused process control and apoptosis-pathway edits to curb lysis-derived impurities. Together, these insights define a practical route to a “Cleaner CHO” chassis.

## Data Availability

The original contributions presented in this study are included in the article and Supplementary Material. Additional underlying data are not publicly available because they contain confidential and proprietary information and are owned by Sartorius Stedim Cellca GmbH, which restricts public release. Requests to access these data should be directed to the corresponding author, Jannis Marzluf (jannis.marzluf@gmail.com), and will be considered upon reasonable request, subject to approval by Sartorius Stedim Cellca GmbH and applicable confidentiality restrictions.
