# Supplementary material for: Towards a cleaner CHO chassis: systematic knockout of host cell proteins for efficient biopharmaceutical manufacturing
Source: Front Bioeng Biotechnol. 2026 Jan 30;14:1750646. doi: 10.3389/fbioe.2026.1750646 (PMC12902299; doi:10.3389/fbioe.2026.1750646)
Supplement: Supplementary file 1 [file DataSheet1.pdf]

Supplemental Information

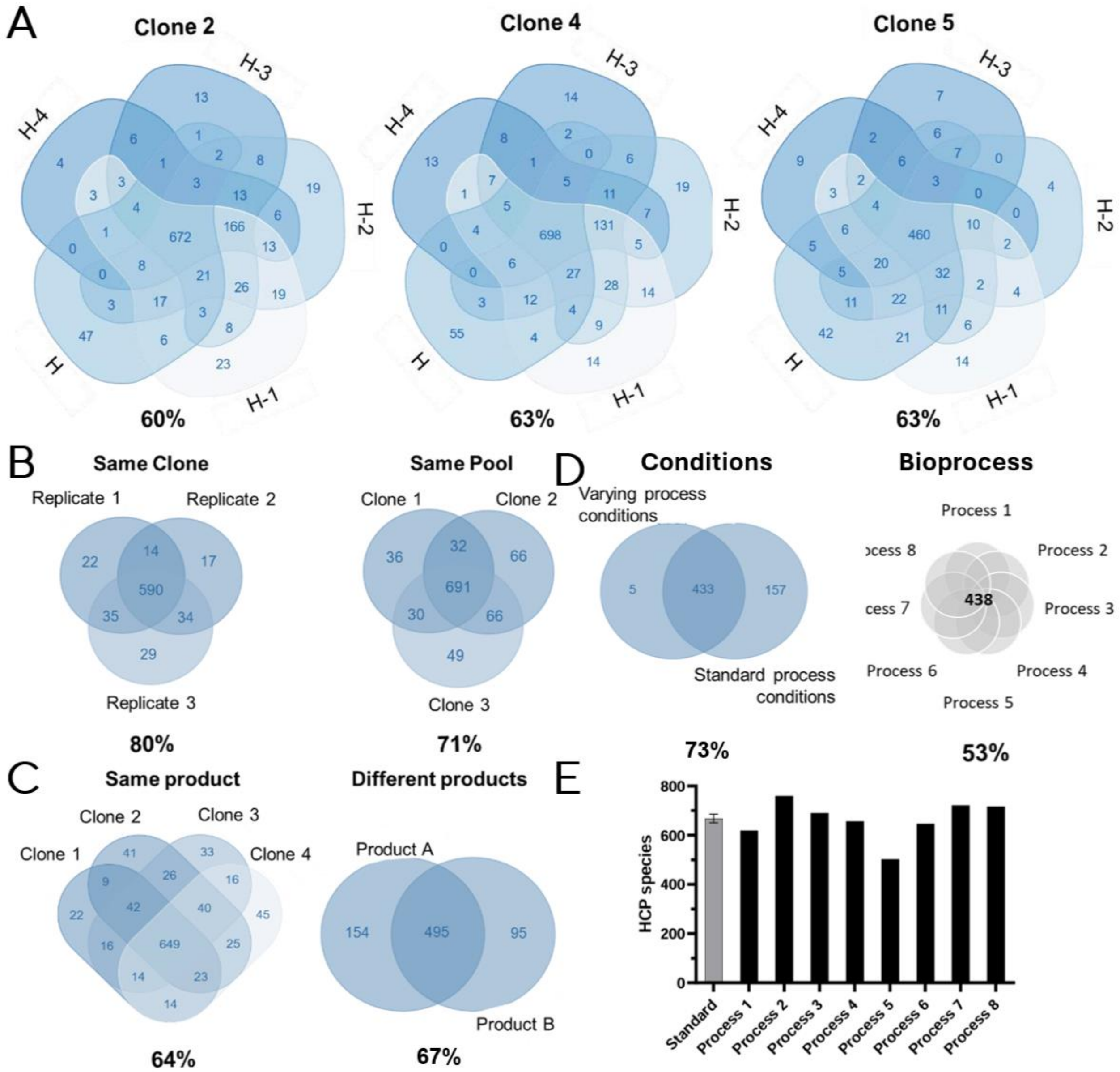

Supplementary Figure 1: ***Overlap Analysis of LC-MS/MS Peptide Mapping Data for abundant HCPs.*** *A: Comparison of HCP species identified at different process days in three individual clones. Percentages represent the number of overlapping HCP species relative to the sum of HCP species being compared. B & C: Comparison of clone origin and product influence on HCP profile similarity. Percentages are overlapping HCP species relative to the sum of HCP species being compared. D: Almost all common HCP species present under the variable conditions are also produced in the standard process. The number of HCP species being shared by cells grown under varying process conditions. E: A comparison of the HCP number produced by a clone under varying and standard process conditions. Error bars are standard deviations. Percentages at the bottom of Venn Diagrams are overlapping HCP species relative to the sum of HCP species being compared.*

General

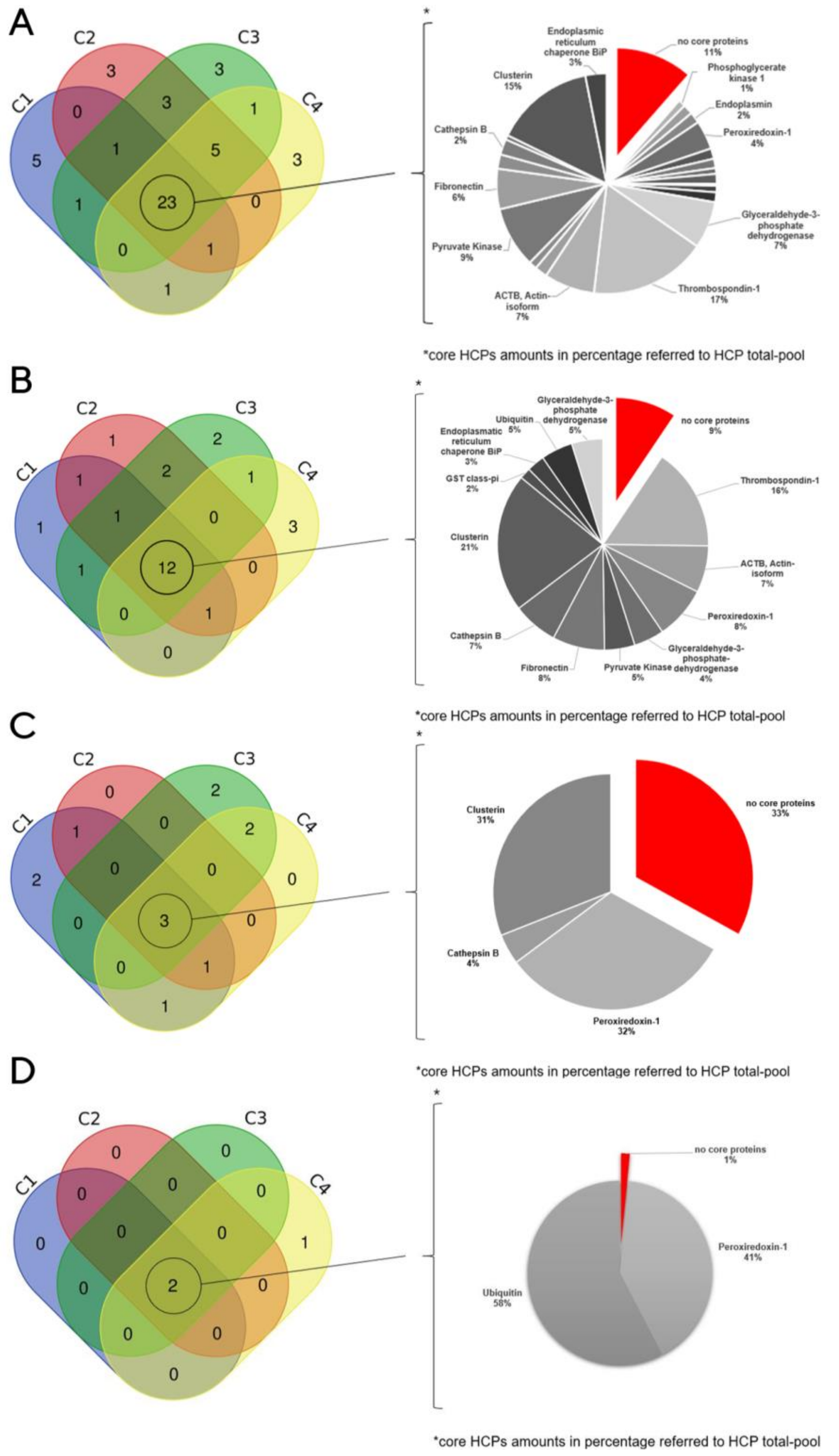

Supplementary Figure 2: ***Overlap Analysis of LC-MS/MS Peptide Mapping Data for difficult-to-remove HCPs.*** *A: HCP-pool after ProtA-chromatography. Identification of core HCPs (proteins present in all four clones C1 - C4) using Venn-Analysis (left) and mass-distribution of those within the samples (right). ProtA = Protein A, C = Clone. B: HCP-pool after virus inactivation. Identification of core HCPs (proteins present in all four clones C1 – C4) using Venn-Analysis (left) and mass-distribution (right) of those within a sample (right). C = Clone. C: HCP-pool after CEX. Identification of core HCPs (proteins present in all four clones C1 – C4) using Venn-Analysis (left) and mass-distribution (right) of those within a sample. CEX = Cation-exchange chromatography, C = Clone. D: HCP-pool after AEX. Identification of core HCPs (proteins present in all four clones C1 – C4) using Venn-Analysis (left) and mass-distribution of those within a sample (right). AEX = Anion-exchange chromatography, C = Clone.*

General

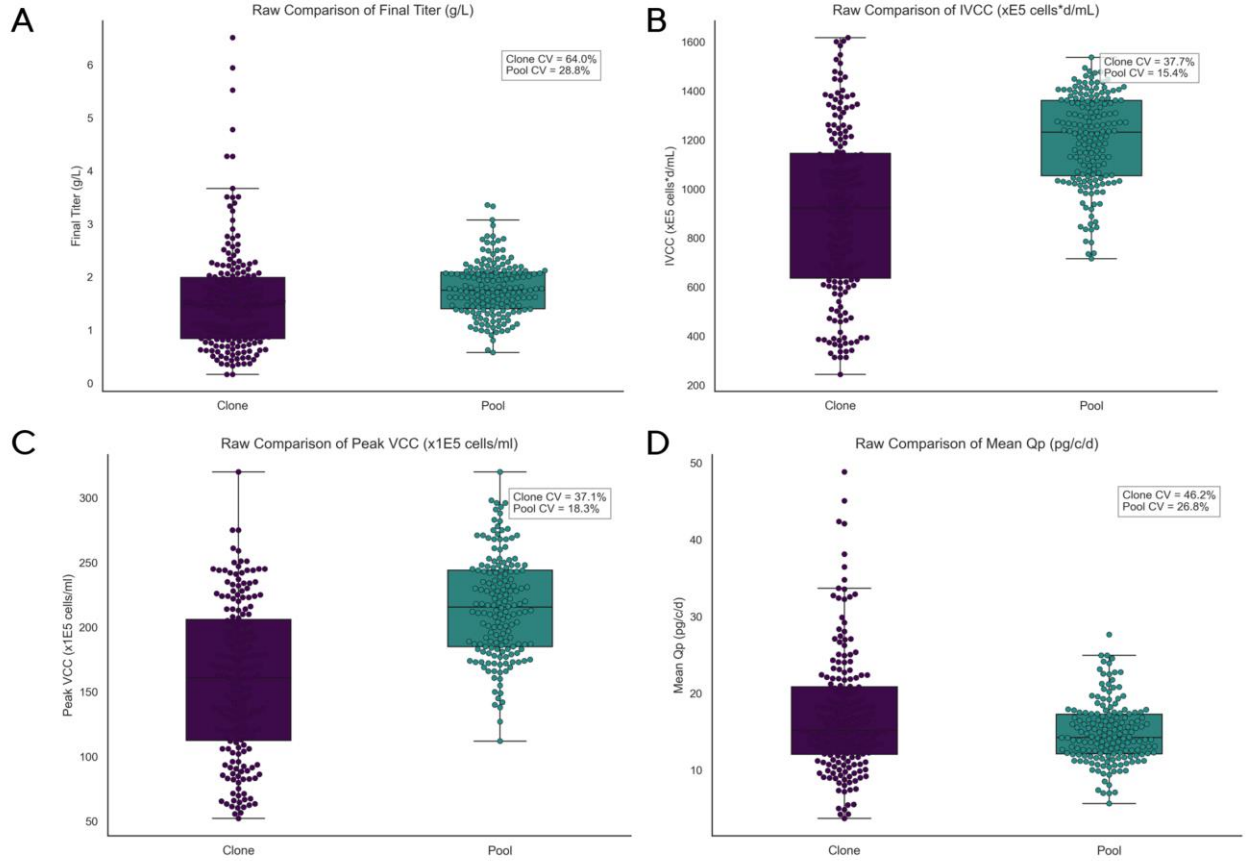

Supplementary Figure 3: ***Comparison of variability between single-cell clones and pooled knockout populations across key bioprocess parameters.*** *Boxplots with overlaid datapoints show raw values for (A) final titer, (B) integral viable cell concentration (IVCC), (C) peak viable cell concentration (VCC), and (D) mean cell-specific productivity (Qp). Clonal datasets displayed higher variability across all four metrics, while pooled knockout populations exhibited reduced variance (coefficients of variation shown in each panel), supporting their use for faster and more consistent evaluation.*

General

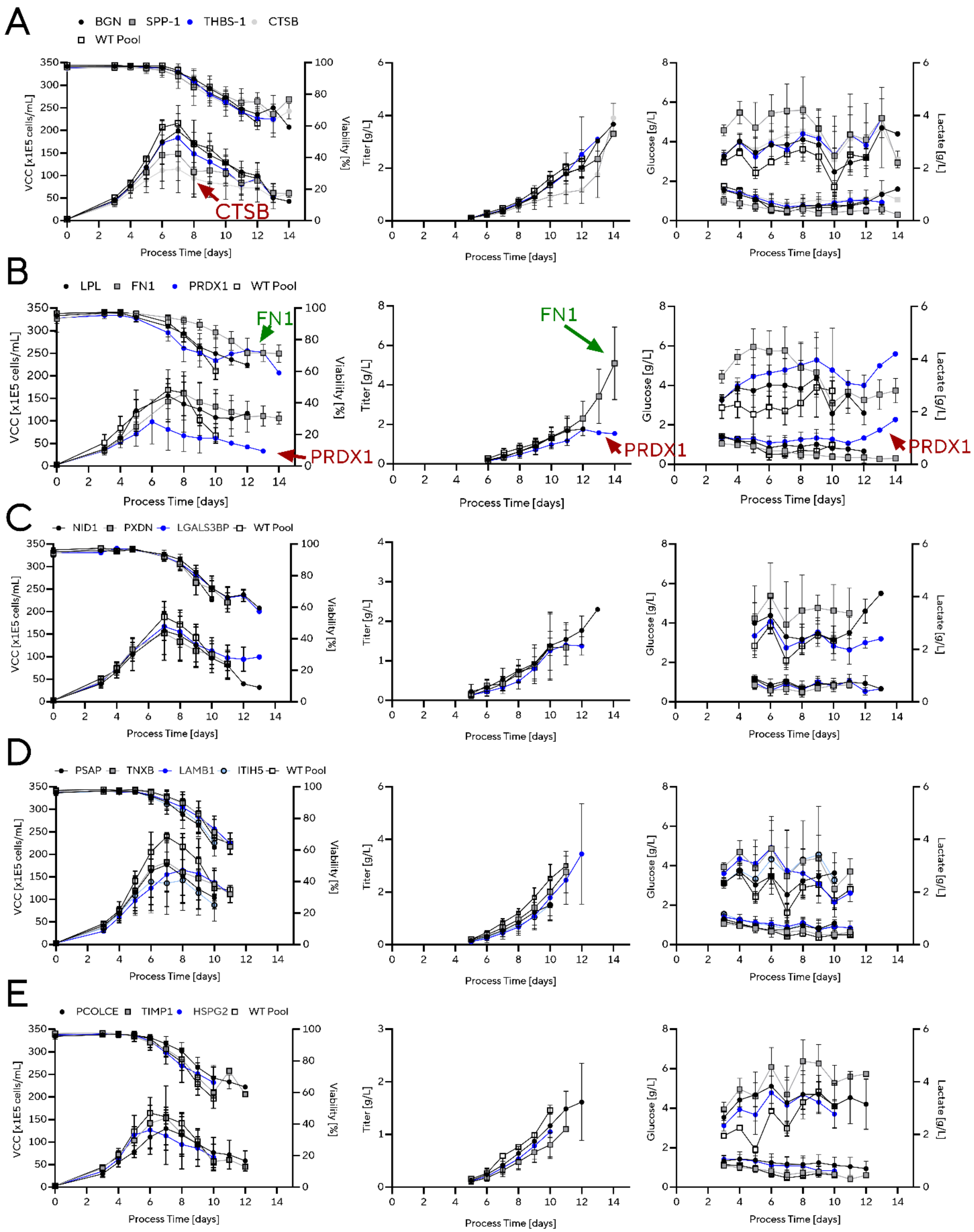

Supplementary Figure 4: ***Fed-batch characterization of the first 17 HCP knockout candidates across clone batches 1–5.*** *Each row represents one batch (A-E), with data shown for (left) viable cell concentration (VCC) and viability, (middle) titer, and (right) glucose and lactate concentrations. Wild-type controls are shown for comparison. Genes with notable*

phenotypes are annotated (e.g., FN1 improved culture longevity and titer; PRDX1 and CTSB reduced growth/productivity). Data are shown as mean ± SD of biological replicates.

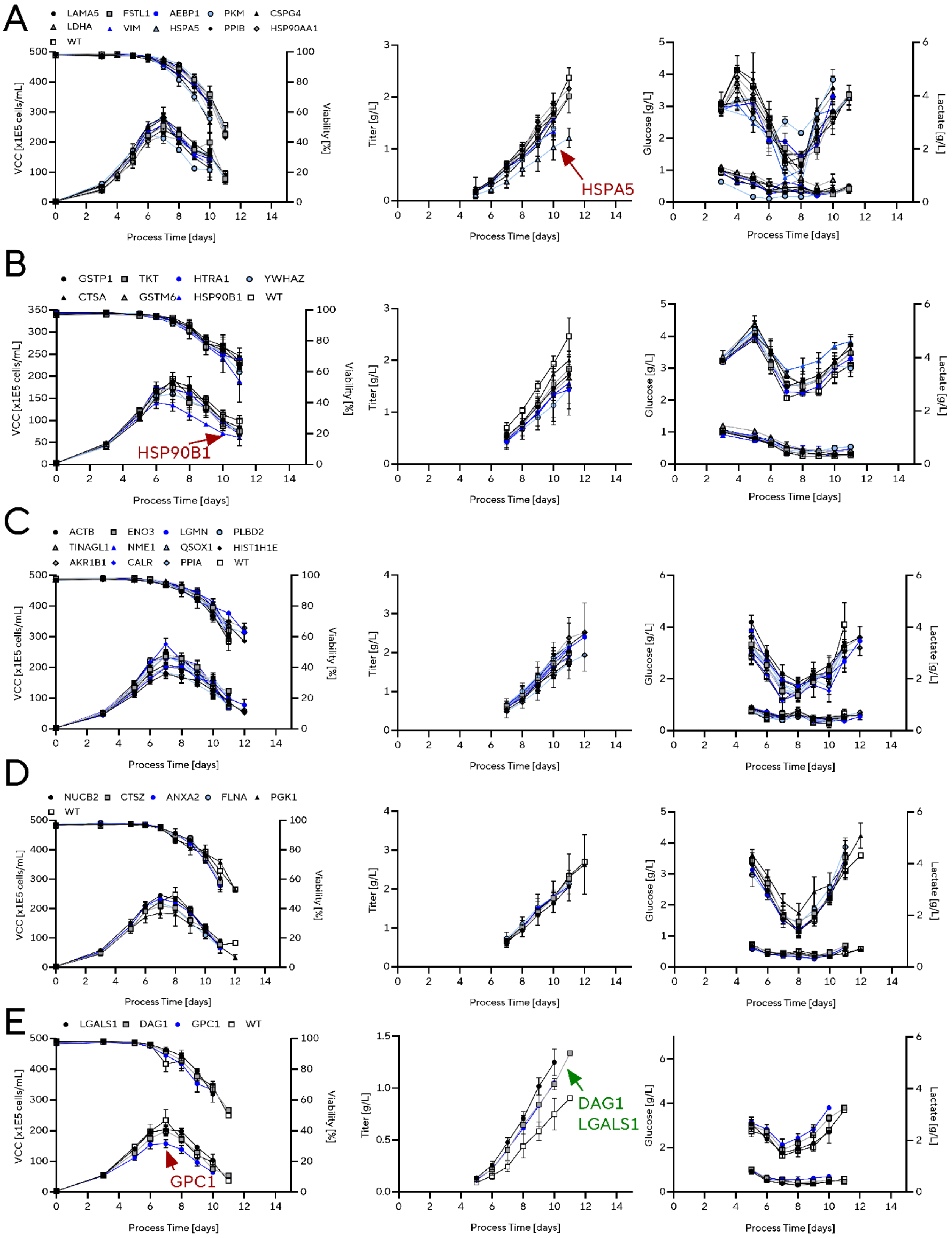

General

Supplementary Figure 5: ***Fed-Batch Bioprocess Characterization of HCP KO Pools (Pool Batches 1-5).*** *Each panel shows viable cell concentration (VCC) and viability (left), titer (middle), and glucose/lactate (right) profiles for a given batch: (A) Batch 1, (B) Batch 2, (C) Batch 3, (D) Batch 4, and (E) Batch 5. Wild-type controls are shown for comparison. Genes with notable phenotypes are annotated (e.g., HSPA5, HSP90B1, GPC1 with reduced performance; DAG1/ LGALS1 showing neutral/beneficial phenotypes). Data are plotted as mean ± SD of biological replicates.*

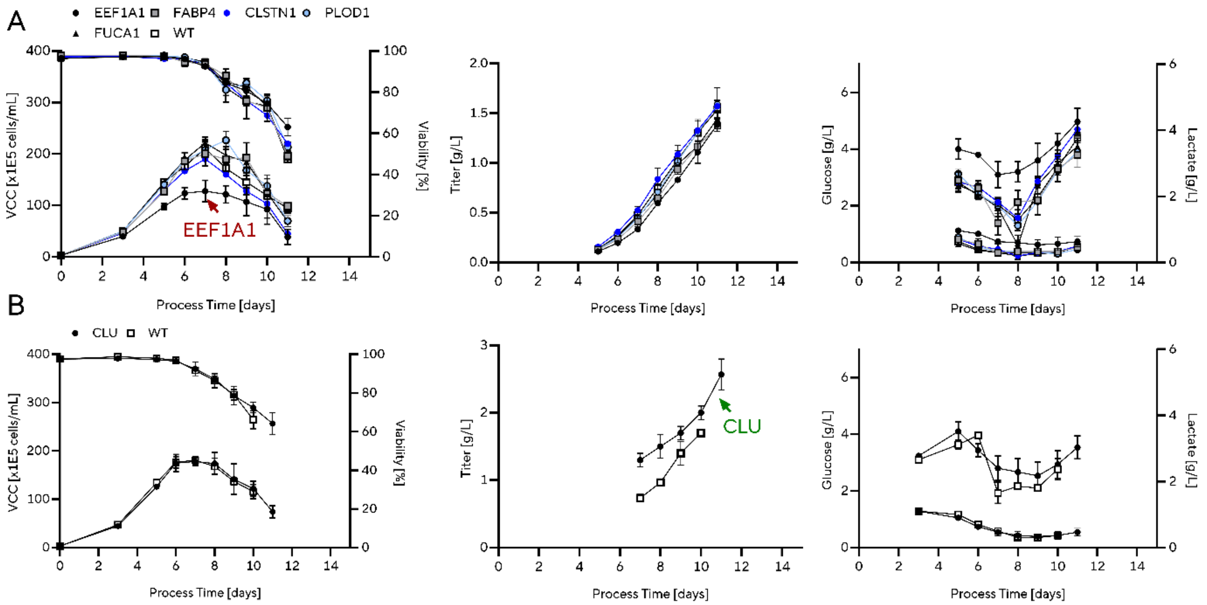

Supplementary Figure 6: ***Fed-Batch Bioprocess Characterization of HCP KO Pools (Pool Batches 6-7).*** *Viable cell concentration (VCC) and viability (left), titer (middle), and glucose/lactate (right) profiles are shown for (A) Batch 6 and (B) Batch 7. Wild-type controls are included for comparison. Genes with notable phenotypes are annotated, including EEF1A1 (reduced performance) and CLU (neutral/beneficial). Data are plotted as mean ± SD of biological replicates.*

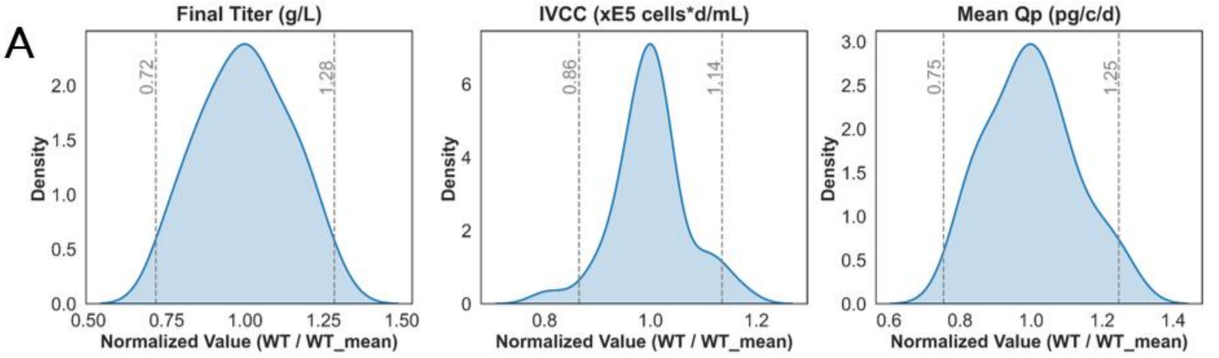

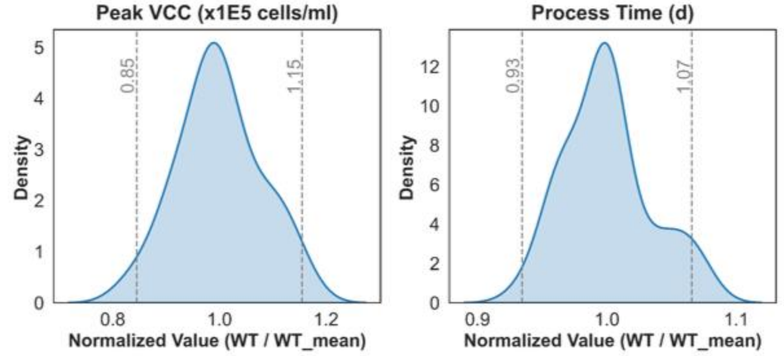

Bioprocess Metric	Lower Cutoff	Upper Cutoff
Peak VCC	0.80	1.20
Process Time	0.92	1.08
Final Titer	0.70	1.3
IVCC	0.80	1.2
Mean Qp	0.70	1.3

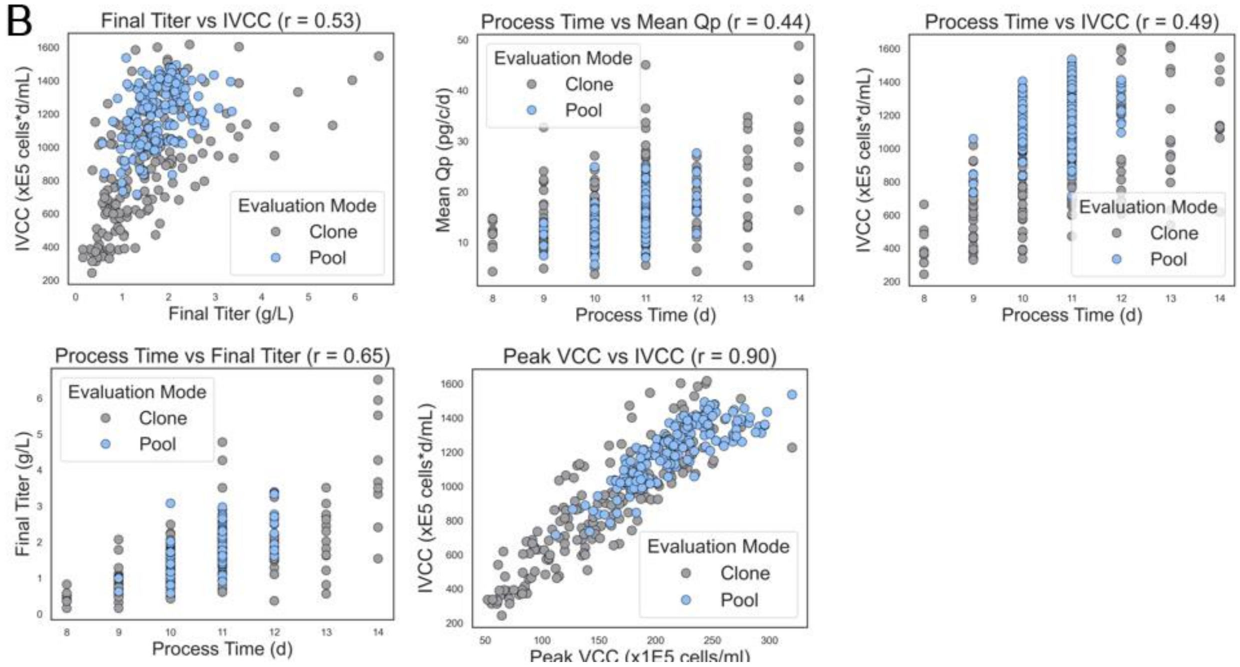

*Supplementary Figure 7: **Correlation analysis of bioprocess parameters across all KO evaluations.** (A) Distribution of normalized process metrics (WT/WT_mean) across clone and pool experiments, including time to peak VCC, peak VCC, process duration, final viability, final titer, IVCC, and mean Qp. Dashed lines indicate ±2×SD thresholds used for performance cutoffs. (B) Pairwise Pearson correlation analysis of key parameters. Strong correlations were observed between peak VCC and IVCC (r = 0.90) and between process duration and final titer (r = 0.65). Final titer correlated moderately with IVCC (r = 0.53) and weakly with peak VCC (r = 0.33). Process duration also correlated with IVCC (r = 0.49) and mean Qp (r = 0.44). These interdependencies are consistent with the expected trade-offs in fed-batch CHO processes, including the weak negative association of peak VCC with mean Qp described in the main text.*
